# Supplementary material for: IL-4, IL-13 and IFN-γ -induced genes in highly purified human neutrophils
Source: Cytokine. Author manuscript; Available in PMC 2023 Jul 10. (PMC10331420; doi:10.1016/j.cyto.2023.156159)
Supplement: supplemental figures/files included [file NIHMS1908116-supplement-supplemental_figures_files_included.pdf]

Cytokine 164 (2023) 156159

Contents lists available at ScienceDirect

Cytokine

journal homepage: www.elsevier.com/locate/cytokine

# IL-4, IL-13 and IFN-γ -induced genes in highly purified human neutrophils

Laura Kummola [a], Tanja Salomaa [b,c], Zsuzsanna Ortutay [d], Ram Savan [e], Howard A. Young [f], Ilkka S. Junttila [b,c,g,h,*]

[a] Biodiversity Interventions for Well-being, Faculty of Medicine and Health Technology, Tampere University, 33014 Tampere, Finland
[b] Cytokine Biology Research Group, Faculty of Medicine and Health Technology, Tampere University, 33014 Tampere, Finland
[c] Fimlab Laboratories, 33520 Tampere, Finland
[d] HiDucator Oy, 36200 Kangasala, Finland
[e] Department of Immunology, School of Medicine, University of Washington, 98195 Seattle, WA, USA
[f] Center for Cancer Research, National Cancer Institute, 21702 Frederick, MD, USA
[g] Northern Finland Laboratory Centre (NordLab), 90220 Oulu, Finland
[h] Research Unit of Biomedicine, University of Oulu, 90570 Oulu, Finland

ARTICLE INFO

*Keywords:*
Neutrophil
IL-4
IL-13
Signal transduction
Allergy
Transcriptome
Type 2 immune response

ABSTRACT

Interleukin (IL)-4 and IL-13 are related cytokines with well-known specific roles in type 2 immune response. However, their effects on neutrophils are not completely understood. For this, we studied human primary neutrophil responses to IL-4 and IL-13. Neutrophils are dose-dependently responsive to both IL-4 and IL-13 as indicated by signal transducer and activator of transcription 6 (STAT6) phosphorylation upon stimulation, with IL-4 being more potent inducer of STAT6. IL-4-, IL-13- and Interferon (IFN)-γ-stimulated gene expression in highly purified human neutrophils induced both overlapping and unique gene expression in highly purified human neutrophils. IL-4 and IL-13 specifically regulate several immune-related genes, including IL-10, tumor necrosis factor (TNF) and leukemia inhibitory factor (LIF), while type1 immune response-related IFN-γ induced gene expression related for example, to intracellular infections. In analysis of neutrophil metabolic responses, oxygen independent glycolysis was specifically regulated by IL-4, but not by IL-13 or IFN-γ, suggesting specific role for type I IL-4 receptor in this process. Our results provide a comprehensive analysis of IL-4, IL-13 and IFN-γ -induced gene expression in neutrophils while also addressing cytokine-mediated metabolic changes in neutrophils.

## 1. Introduction

Neutrophilic granulocytes, neutrophils, are the most abundant type of circulating white blood cells in humans. These polymorphonuclear phagocytes form a critical part of body's first-line defense against invading pathogens; they are rapidly recruited to the site of microbial invasion, where they utilize their diverse toolbox of defense mechanisms to eradicate the intruder [1]. In addition to phagocytosis, these defensive mechanisms include the release of antimicrobial agents by degranulation, production of reactive oxygen species and formation of neutrophil extracellular traps (NETs) [2]. Neutrophils also secrete cytokines and chemokines that attract other immune cells, resulting in the amplification of the immune response [3–5].

Thus, neutrophils are a multifunctional cell population equipped with the ability to phagocytose invading microbes and modulate immune responses [6]. For example, neutrophils can secrete IL-10 [7,8], and they express or release immunomodulatory molecules, such as programmed death ligand 1 (PD-L1) [9–11] and Arginase 1 [12,13]. Neutrophils can also modify dendritic cell functions [14,15]. In the context of cancer, they have both anti- and pro-tumorigenic functions [16,17].

The role of neutrophils in type 2 immune response is intriguing. They

*Abbreviations:* AnxA1, annexin A1; EC50, effective concentration 50 %; ECAR, extracellular acidification rate; HRH4, histamine H4 receptor; IFN, interferon; IL, interleukin; IRS2, insulin receptor substrate 2; KEGG, Kyoto Encyclopedia of Genes and Genomes; LIF, Leukemia inhibitory factor; NET, neutrophil extracellular trap; OCR, oxygen consumption rate; PCA, principal component analysis; PD-L, programmed death ligand; PGD, phosphogluconate dehydrogenase; PPERT, probability of a signaling perturbation of a gene; PPP, pentose phosphate pathway; PTX3, pentraxin 3; RotA/A, Rotenone A and Antimycin; SPIA, signaling pathway impact analysis; STAT, signal transducer and activator of transcription; TNF, tumor necrosis factor.

* Corresponding author at: Faculty of Medicine and Health Technology, Tampere University, Arvo building Rm F326, Arvo Ylpönkatu 34, 33520 Tampere, Finland.
*E-mail address:* ilkka.junttila@tuni.fi (I.S. Junttila).

https://doi.org/10.1016/j.cyto.2023.156159
Received 29 November 2022; Received in revised form 27 January 2023; Accepted 10 February 2023
Available online 19 February 2023
1043-4666/© 2023 The Author(s). Published by Elsevier Ltd. This is an open access article under the CC BY license (http://creativecommons.org/licenses/by/4.0/).

seem to be absent in some settings of atopic disorders, such as atopic dermatitis [18] while their presence has also been associated with inflammation severity [19,20] and late phase reactions [21]. During the early phase of helminth infection, neutrophils take part in the containment of the parasite in murine models [22–24] and they have been shown to promote alternatively activated macrophage polarization during nematode infection [25]. An influx of neutrophils is seen during allergenic challenges in asthma and virus-induced asthma exacerbations [26].

Closely related IL-4 and IL-13 cytokines induce key events of type 2 inflammation such as differentiation of T helper 2 cells (Th2), immunoglobulin E production by B cells, macrophage polarization, eosinophil recruitment and mucus production by goblet cells [27,28]. IL-4 signals through IL-4Rα, a receptor subunit able to complex with the common gamma chain (γc) (type I IL-4R) or with IL-13Rα1 (type II IL-4R) while IL-13 signals only through type II IL-4R. This complexity is important to keep in mind when evaluating the roles of IL-4 and IL-13 in mouse models lacking IL-4, IL-4Rα, IL-13 or IL-13Rα1 expression [29].

Our approach to understand the complex role of neutrophils in type 2 immune response was to directly assess cellular responses to IL-4 and IL-13. To this end, we stimulated primary human neutrophils with IL-4 and IL-13 and measured STAT6 phosphorylation. We used RNA sequencing to determine how the cytokine stimulus affects gene expression. We also examined whether the cytokines have an effect on neutrophil glycolysis and oxygen consumption.

## 2. Materials and methods

### 2.1. Neutrophil isolation and cell culture

Human neutrophils were purified from the peripheral blood of healthy donors with no known allergies, under the ethical permit of Pirkanmaa Hospital District Ethics Committee (permit number R12002). Blood was collected into Na-citrate vacuum tubes (BD, Franklin Lakes, NJ, US) and erythrocyte sedimentation was performed using 3 % dextran in 0.9 % NaCl for 30 min. The resulting plasma was centrifuged, and the pellet was resuspended into HBSS without calcium or magnesium (Lonza, Basel, Switzerland). Cell suspension was layered over Histopaque®-1077 (Sigma-Aldrich, Saint Louis, MO, US) and neutrophils were separated from mononuclear cells with gradient centrifugation. A 30-second hypotonic lysis of remaining red blood cells was done with 0,2 % and 1,6 % NaCl solutions twice. For RNA sequencing, neutrophils were isolated using a discontinuous Percoll-gradient method as described previously [30], followed by sorting (see section *2.2 Flow Cytometry*). The Percoll method might result in less priming of the neutrophils [30] (and thus was selected for the RNASeq), but in our hands was more inconsistent in terms of neutrophil yield, so for other assays we chose the Histopaque-method. After the isolation, neutrophils were resuspended to a concentration of 1-4x$10^6$ cells/ml into RPMI 1640 with 10 % FBS, L-glutamine and penicillin/streptomycin (all Lonza) and stimulated with 50 or 100 ng/ml IL-4, IL-13 or IFN-γ (Peprotech, Cranbury, NJ, US) at 37°C for subsequent analyses. The stimulation time depended on the assay to be performed (see sections below).

### 2.2. Flow cytometry

Phospho-STAT analysis was performed on freshly drawn venous blood from self-reportedly healthy, non-allergic donors (n = 6). Briefly, whole blood was surface stained for CD16 and CD11b and simultaneously stimulated with IL-4 or IL-13 (100, 10, 1, 0.1 and 0.01 ng/ml) or IFN-γ (100 ng/ml) for 15 min at 37°C. Red blood cells were lysed with BD Phosflow™ Lyse/Fix Buffer, permeabilized with −20°C 90 % methanol in PBS and incubated overnight at −20°C. After this, the cells were stained with anti-pSTAT6 or anti-pSTAT1. The samples were analyzed with FACSCanto II (BD). FlowJo software (Tree Star, Ashland, OR, US) was used for further data analysis.

For RNASeq, isolated neutrophils (n = 4) (see *Neutrophil Isolation and Cell Culture* and *RNASeq*) were sorted with BD FACSAria Fusion to remove contaminating cells. To avoid neutrophil activation, an antibody-free, no-stain protocol was used [31]. Neutrophils were identified based on forward and side scatter characteristics and low autofluorescence on a 525/50 nm bandpass filter of the 405 nm laser line.

Surface staining of neutrophils was performed to determine the expression of PD-L1 and PD-L2 in neutrophil subsets and to assess neutrophil purity after sorting.

Antibody and panel details are listed in Supplementary Table 1.

### 2.3. RNASeq

For mRNA sequencing, the RNA from neutrophils were extracted using RNeasy Mini Kit and RNase-Free DNase Set (Qiagen, Hilden, Germany). The preparation of the RNA libraries and sequencing was carried out in the Finnish Functional Genomics Centre (Turku, Finland). The RNA libraries were prepared according to the Illumina Stranded mRNA Preparation kit (San Diego, CA, US) protocol 1,000,000,124,518 using Human Brain Total RNA AM7962 (ThermoFisher Scientific, Waltham, MA, US) as a positive control. The quality of the RNA and later RNA libraries were ensured using Agilent Advanced Analytical Fragment Analyzer (Santa Clara, CA, US) and sample concentration was measured with Qubit® Fluorometric Quantitation (Life Technologies, Carlsbad, CA, US). Sequencing was performed using Illumina NovoSeq 6000 S4 V1.5 and 2 × 100 bp read lengths.

The quality of the sequenced reads was assessed using FastQ (v.0.11.8) [32] and MultiQC (v.1.10) [33] software with default parameters. Reads were aligned to the human genome (hg38) with Rsubread (v.2.0.0) aligner [34]. Statistical analysis of differential gene expression was assessed by DESeq2 algorithms [35], available at [36]. Data was analyzed in pairs, comparing read counts of cytokine stimulated cells to their unstimulated counterparts. We considered genes as differentially expressed when the Benjamini–Hochberg adjusted p-value was$<$0.05.

For gene set enrichment analyses, genes were ordered based on fold change values (log2FC) and gene set enrichment was analyzed using EnrichR [37,38].

Signaling pathway impact analysis was implemented according to Tarca et. al. [39] using the SPIA R package 2.48.0, available at [40].

Clustered heatmap was generated from shrinked gene expression values [41] by using the pheatmap package (v.1.0.12.), available at [42], in the R software.

### 2.4. Glycolytic rate assay

To study cytokine impact on neutrophil glycolysis and oxygen consumption, real-time extracellular acidification rate (ECAR) and oxygen consumption rate (OCR) were measured using Glycolytic rate assay kit with XFe24 Seahorse Analyzer (Agilent). After stimulation with IL-4, IL-13, IFN-γ (50 ng/ml for 1 h) or medium only, human peripheral neutrophils from healthy donors (n = 6) were washed twice with RPMI 1640 with supplements and resuspended into prewarmed Seahorse XF DMEM medium containing 2 mM glutamine, 10 mM glucose and 1 M pyruvate (Agilent). 50 µl of 50 µg/ml Poly-D-lysine (Life Technologies) in PBS was used for coating 24 microplate wells. The plate was incubated for 1 h at RT and then washed twice with sterile $H_2O$ and let dry completely. 1 × $10^6$ neutrophils in 100 µl of Seahorse assay medium were added to each well and the plate was centrifuged at 200*g* (zero brake) for 1 min to attach the neutrophils on the bottom. The plate was incubated at 37 °C in a non-$CO_2$ incubator for 25 min. 400 µl of assay medium was gently added to each well and incubated for 30 min to settle the conditions and $CO_2$ levels prior to the analysis. For the analysis, the Seahorse cartridge was loaded with rotenone A and antimycin (Rot A/A) at 10× the final concentration of 0,5 µM in port A and 2-deoxy-D-glucose (2-DG) at 10×

final concentration of 50 mM in port B. Six independent experiments were performed with 3–4 independent replicates per group. Results were analyzed using Wave version 2.6 (Agilent).

### 2.5. Statistical analysis

All data were analyzed with Prism 9 software (GraphPad software, La Jolla California, USA). All data were considered statistically significant for P values<0.05. Statistical tests are indicated in figure legends.

## 3. Results

### 3.1. IL-4 and IL-13 induce pSTAT6 in human neutrophils

Whole blood from healthy volunteers (n = 6) was stimulated with IL-4 and IL-13 or left unstimulated for 15 min and the phosphorylation of tyrosine (Y) 641 of STAT6 in $CD11b^{high}CD16^{high}$ cells was measured by flow cytometry (Fig. 1A). We ran a titration series of rising concentration of IL-4 and IL-13 and additionally confirmed the phosphorylation of Y701 STAT1 in response to IFN-γ. (Fig. 1B-D). A clear induction of pSTAT6 was seen with a concentration as low as 0.01 ng/ml of IL-4 (p = 0.007) and a maximum response was reached with a concentration of 1 ng/ml of IL-4 (p = 0.001). Neutrophils were able to respond to IL-4 with a concentration ten times less than the minimum responsive dose for IL-13; p-values for different stimulations are provided in Supplementary Table 1. In line with this, the half maximal effective concentration value ($EC_{50}$) for IL-4 was 0.041 ng/ml and for IL-13 0.435 ng/ml ($p < 0.0001$). We also enriched neutrophils from the blood, bone marrow and spleen of transgenic *IL-22R1* mice that develop spontaneous neutrophilia [43] and subjected the cells to stimulation with IL-4 and IL-13 for 15 min. Flow cytometric analysis of Gr-1 + neutrophils showed a statistically significant induction of pSTAT6 with IL-4 in the bone marrow and spleen (Supplementary Fig. 1A).

### 3.2. IL-4-, IL-13- and IFN-γ regulated gene expression in human neutrophils

Next, we identified target genes of IL-4 and IL-13 in human neutrophils. We performed an RNA sequencing analysis of neutrophils isolated from four healthy donors. To minimize the contamination of neutrophils

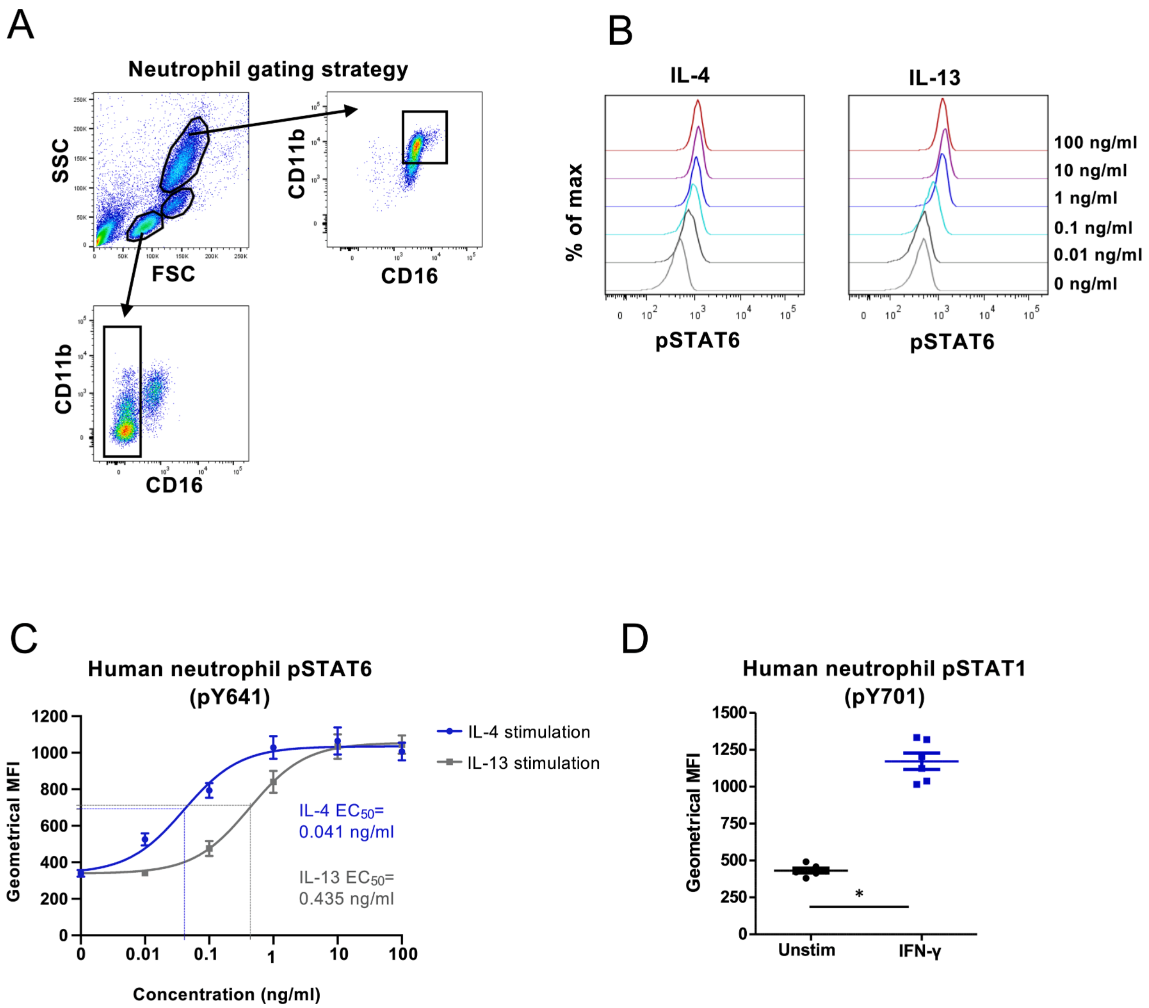


**Fig. 1. Responsiveness of human neutrophils to 15 min stimulation with IL-4, IL-13 and IFN-γ, measured by phosphorylation of STAT6 and STAT1 using flow cytometry.** (A) Gating strategy for neutrophils, monocytes and lymphocytes. Neutrophils were defined as $CD11b^{high}$ $CD16^{high}$ cells. Monocytes and lymphocytes were gated based on forward and side scatter characteristics and CD16 + cells were excluded from cells in lymphocyte gate. Doublets were excluded from all three populations (not shown). (B) Stimulation of human whole blood was performed with indicated amounts of IL-4 or IL-13 and phosphorylation of STAT6 was determined in $CD11b^{hig}hCD16^{high}$ cells. Representative histograms of fluorescence intensity are shown. (C) Geometrical means ± SEM of pSTAT6 fluorescence of six donors after stimulation with IL-4 or IL-13. The curves of both stimulations are fitted using non-linear regression 3-parameter fit (goodness of fit was evaluated with R square and the values were for IL-4 and IL-13 0.8604 and 0.8961, respectively) and $EC_{50}$ values are indicated based on the fitted curves. The IL-4 10 ng/ml datapoint was lost for one donor. (D) Geometrical means of pSTAT1 (pY701) fluorescence of six donors after whole blood was either left untreated or stimulated with 100 ng/ml of IFN-γ for 15 min, *) p = 0,0313; Wilcoxon matched-pairs signed rank test.

with eosinophils or monocytes, the cells were first enriched with discontinuous Percoll gradient and subsequently sorted with flow cytometry (Supplementary Fig. 1B-C). The purified cells were subsequently stimulated with 100 ng/ml of IL-4, IL-13 or IFN-γ for 1 h and the RNA was extracted and subjected to RNASeq analysis. Differentially expressed genes were assessed using DESeq2 [35]. Fig. 2A presents a Venn diagram of the number of IL-4, IL-13 and IFN-γ induced transcripts and Fig. 2B shows the principal component analysis (PCA) of the genes. We identified 330, 318 and 261 up- or downregulated genes for IL-4, IL-13 and IFN-γ, respectively, with an adjusted p-value under 0.05. Principal component analysis confirms that IL-4 and IL-13 stimulations lead to highly similar changes in gene expression while treating neutrophils

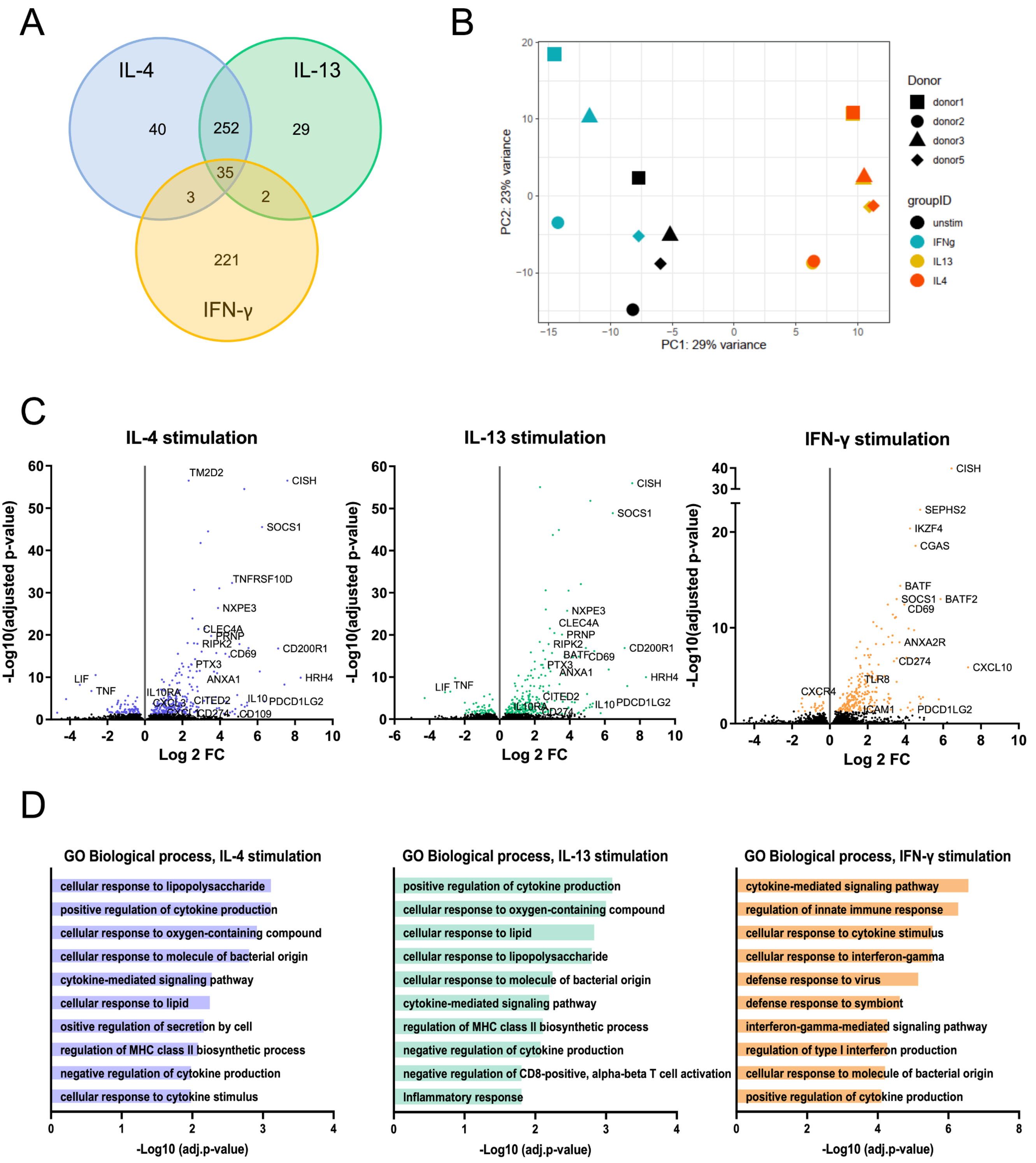


**Fig. 2. RNA sequencing analysis of up- and downregulated genes in human neutrophils after stimulation with IL-4, IL-13 or IFN-γ.** (A) Venn diagram of genes regulated in human neutrophils after different stimulations. (B) Principal component analysis of stimulations and donors. (C) Volcano plots of IL-4-, IL-13- and IFN-γ-regulated genes in human neutrophils. Genes with a cutoff of false discovery rate of 5 % are shown (colored dots). (D) Genes up- and downregulated upon stimulation with IL-4, IL-13 or IFN-γ were analyzed using Enrichr. Ten Gene Ontology biological processes with the smallest adjusted p-values are shown for each stimulation. Fisher exact test was used for the calculation of p-values and correction for multiple comparisons was performed with the Benjamini-Hochberg method.

with IFN-γ results changes in the expression patterns of a different set of genes. While the major discriminatory factor is the cytokine stimulus, the gene expression pattern is affected almost equally by the biological/genetical background of the donors. For IL-4 and IL-13 stimulations, most genes were overlapping. Volcano plots provided in Fig. 2C visualize the differential gene expression by different stimulations. Certain immune-related genes are highlighted.

Both IL4 and IL-13 downregulate *LIF* and *TNF* whereas genes coding for *CD200R1*, pentraxin 3 (*PTX3*), annexin A1 (*AnxA1*), histamine H4 receptor (*HRH4*), *IL-10, PD-L1* (*CD274*) and *PD-L2* (*PDCD1LG2, CD273*) were up-regulated. IFN-γ induces specific set of genes, many of which are logically related to intracellular infections. General comparison of ten Gene Ontology biological processes with the smallest adjusted p-values regulated by IL-4, IL-13 or IFN-γ are shown in Fig. 2D. IL-4 and IL-13 regulated genes seem to be connected to mostly the same biological processes, including regulation of cytokine production and regulation of MHCII biosynthetic process and cellular responses to oxygen and lipopolysaccharide. Processes connected to IFN-γ stimulation, on the other hand, seem to be related to innate responses and viral infections. The analysis was done using Enrichr data analysis tool [37,38]. To gain insight into the biological relevance of the changes in gene expression following cytokine stimuli, we aimed to identify affected signaling pathways. We employed the signaling pathway impact analysis (SPIA), which combines the classical over-representation analysis of differentially expressed (DE) genes of a Kyoto Encyclopedia of Genes and Genomes (KEGG) pathway with the perturbation of signal transduction caused by these genes on the given pathway under the experimental condition [39]. SPIA takes into consideration the probability of obtaining at least as much DE genes on the given pathway as is observed in the experimental setup (PNDE) and at the same time, the probability of a signaling perturbation of a gene (PPERT), meaning its average expression difference between two conditions and the influence of upstream genes on it. Not surprisingly, the ten most significantly affected pathways were common following IL-4 or IL-13 stimulus although with different statistical probability (Fig. 3A), while IFN-γ affected the activity of a mostly different set of pathways.

Fig. 3B shows heatmap representation of chosen genes further confirming that IL-4 and IL-13 regulates mostly the same genes while IFN-γ induce a separate set of genes. Most IL-4 induced genes were also induced by IL-13, suggesting that type II IL-4R in neutrophils can drive same genes independent of the ligand triggering the receptor recruitment. The complete dataset can be accessed at Gene Expression Omnibus (GEO) accession number GSE218535 and the set of genes with adjusted p-value under 0.05 is shown in Supplementary Table 2.

### 3.3. PD-L2 expression in neutrophils

One of the genes induced by all three cytokines was *PDCD1LG2*, which codes for PD-L2. This is a ligand of T cell PD-1 receptor (CD279) and a member of checkpoint inhibitor molecules. In cancer, the expression of PD-L2 on cancer cells can downmodulate antitumor responses [44]. In murine splenic cells, PD-L2 expression was inducible by both IL-4 and IFN-γ in macrophages and dendritic cells [45] as in our RNASeq data.

In humans, allergic inflammation reduces the expression of PD-L2 in myeloid dendritic cells [46]. We first compared PD-L2 expression in IL-4 and IL-13 stimulated neutrophils by flow cytometry. Freshly isolated neutrophils express high levels of CD16, CD11b and CD66b, but after 20 h of incubation, most neutrophils from healthy donors ($n = 4$) exhibited dramatically decreased levels of CD16 and CD11b. Interestingly, PD-L2 was upregulated in both IL-4 and IL-13 stimulated neutrophils in a population that remained positive for CD16 and CD11b. This upregulation was prominent especially in the IL-4 stimulated cells, whereas unstimulated CD16 + CD11b + neutrophils had low or no expression of PD-L2. These PD-L2 + CD16 + CD11b + cells were also positive for CD66b and CD62L. (Fig. 4A, gating strategy in Supplementary Fig. 2A. Expression of CD16 and CD62L imply an inactive state for neutrophils [47,48].

We also examined the expression of PD-L2 specifically in $CD16^{high}CD62L^{dim}$ neutrophils, which are known suppressors of T cell proliferation via reactive oxygen species (ROS) production [49] and PD-L1 [10]. However, in this "regulatory" neutrophil population PD-L2 was upregulated in only two out of four donors after stimulation with IL-4 or IL-13 (data not shown). Additionally, PD-L1 expression was measured after stimulation, but surprisingly in contrast to RNASeq data, neutrophils were consistently negative for it in our flow cytometric analysis (Supplementary Fig. 2).

### 3.4. IL-4 decreases mitochondria-dependent glycolysis

During inflammatory response, cell activation leads to increased energy consumption. Thus, glycolytic ATP production is increased upon immune cell activation [50]. In neutrophils, mitochondrial number per cell is low, and it has been suggested that mitochondria in neutrophils plays no role in energy production but that they are critical for neutrophil apoptosis [51]. To measure possible cytokine impact on glycolysis and oxygen consumption in neutrophils, Seahorse XFe analyzer was utilized.

Glycolytic rate assay test was performed based on the manufacturer's standard assay (Fig. 5A, Supplementary Fig. 3A-B). The extracellular acidification rate (ECAR), which indicates the anaerobic glycolysis and oxygen consumption rate (OCR) indicating mitochondrial aerobic respiration were measured. After recording the basal levels of ECAR and OCR, mitochondrial respiration chain was inhibited in complexes I and III (Rot/AA) to determine compensatory glycolysis level. Subsequently, glycolysis was blocked completely by 2-DG. The interval between each ECAR and OCR measurements was 8 min 30 sec. There was no significant difference in basal level of glycolysis (ECAR) (Fig. 5B) or oxygen consumption during mitochondrial respiration (OCR) when neutrophils were left untreated or pretreated with indicated cytokines for one hour (Supplementary Fig. 3B). Interestingly, when mitochondrial respiration was inhibited by Rot/AA, the ability of the IL-4 stimulated neutrophils to drive compensatory glycolysis to meet the cells' energy demands was decreased compared to unstimulated cells ($p < 0.045$, Fig. 5C). The OCR in IL-4 stimulated cells also remained at lower level after 2-DG injection compared to unstimulated cells (Supplementary Fig. 3B), albeit this was not statistically significant. Taken together, these results indicate that IL-4 decreases the anaerobic glycolysis in neutrophils. As an aside, quite interestingly Rot/AA injection led to dropped OCR levels under all stimulation conditions studied (Supplementary Fig. 3B), paving way for further experiments on the role of mitochondria in neutrophil ATP production.

## 4. Discussion

Neutrophils are the most abundant leukocytes in blood, in humans constituting 40–70 percent of the total leukocytes. With most half-life estimates ranging from 4 to 19 h [52] their biology appears quite different from other blood leukocytes (or lymphocytes) with substantially longer half-lives. The short half-life of neutrophils both *in vivo* and *in vitro* and their extreme sensitivity to changes in physical conditions in their surroundings hampers studying them. Here, we chose to study direct effects of cytokines on these cells *in vitro*. Obviously, such approach teaches us little on possible feedback loops neutrophils initiate *in vivo* [53].

We discovered significant difference in neutrophil responsiveness to IL-4 and IL-13 as measured by STAT6 activation, similar to what was previously observed in monocytes [54]. This is also in line with the notion that IL-4 protein level is difficult to measure in biologic fluids as compared to IL-13 and thus, higher potency of IL-4 could explain its lower protein levels. It is of interest that high IL-4 levels could lead to harmful side effects as observed in humans [55] or even toxicity as

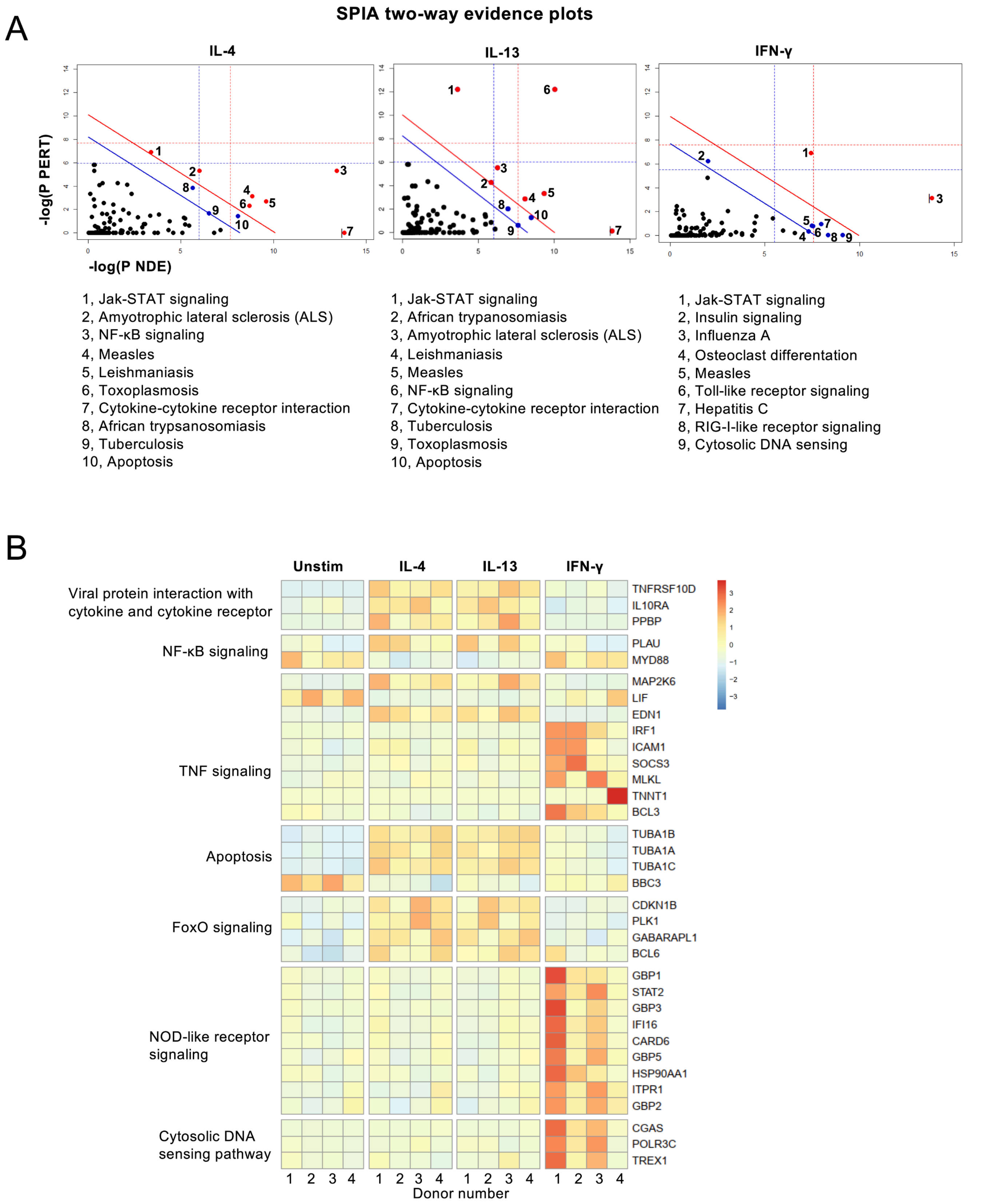


**Fig. 3. Analysis of differentially expressed genes.** Messenger RNA of unstimulated, IL-4, IL-13 or IFN-γ stimulated neutrophils was analyzed using RNASeq. Differential gene expressions were tested applying DESeq2. (A) Signaling pathway impact analysis (SPIA). Two-dimensional plots illustrating the relationship between the two types of evidence considered by SPIA. The X-axis shows the over-representation evidence, while the Y-axis shows the perturbation evidence. Dots represent KEGG pathways. Red dots mark pathways significant at 5 % after Bonferroni correction, blue dots mark significantly enriched pathways ($p < 0.05$) according to FDR correction. (B) Heatmap of differentially expressed genes. Shown genes were selected based on differential expression ($p < 0.05$) and occurrence in only one KEGG pathway. (For interpretation of the references to colour in this figure legend, the reader is referred to the web version of this article.)

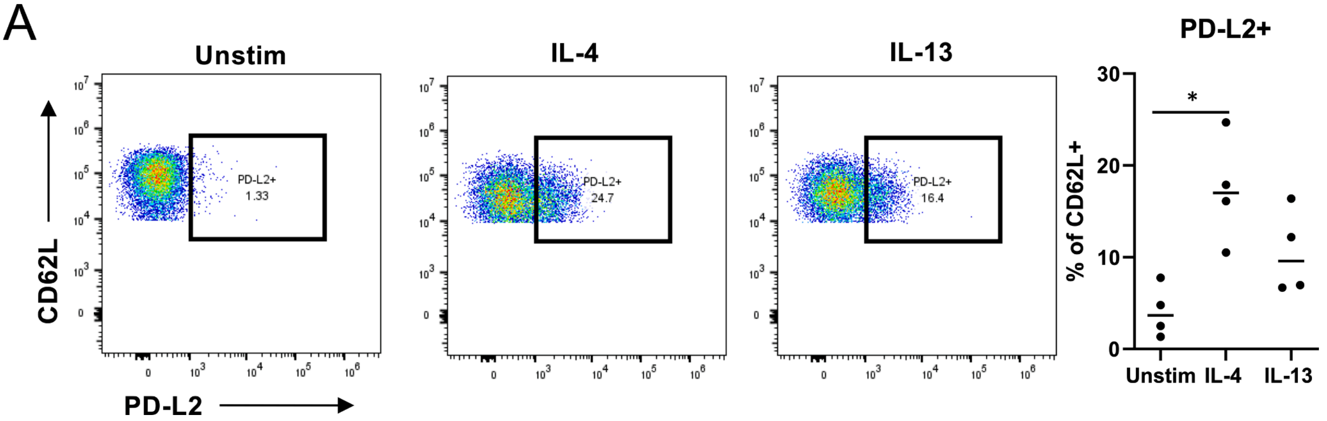


**Fig. 4. PD-L2 expression in IL-4 and IL-13 stimulated human neutrophils.** (A) Neutrophils were stimulated with IL-4 or IL-13 for 20 h and PD-L2 expression was measured in CD16$^{high}$CD11b + CD66b + CD62L$^{high}$ cells with flow cytometry. *) p = 0.0140, Friedman test with Dunn's multiple comparisons test.

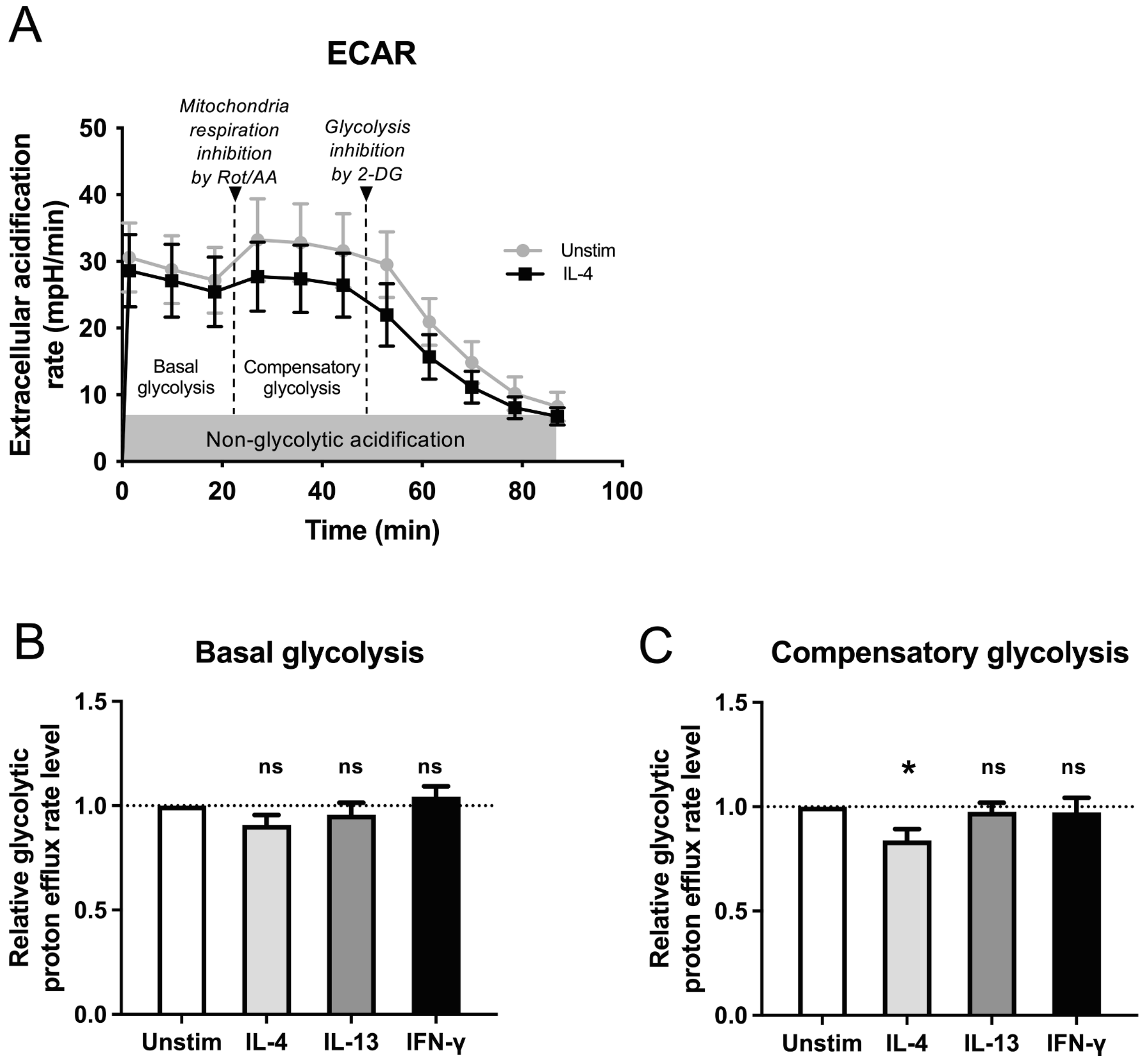


**Fig. 5. Effect of IL-4, IL-13 and IFN-γ on neutrophil energetics.** (A) Extracellular acidification (ECAR) was measured in milli-pH per minute (mpH/min) for 11 cycles. After measuring 3 basal level measurements, 3 measurements were recorded to determine the effect of Rotenone and Antimycin A (Rot/AA) for inhibiting mitochondrial respiration. Additional 5 measurements after injection of 2-deoxyglucose (2-DG) for glycolysis inhibition were detected. Used interval time between measurements was 8 min 30 sec. Representative curve for unstimulated and IL-4 stimulated neutrophils from one experiment is shown. (B) Comparison of relative basal glycolysis of neutrophils that were left unstimulated or stimulated with 50 ng/ml of IL-4, IL-13 or IFN-γ for one hour. The medians of each stimulation in each experiment (n = 6) are compared to unstimulated sample median of the same experiment. Averages and ± SEM are indicated. (C) Comparison of compensatory glycolysis from same samples as in 5B. The medians of each stimulation in each experiment (n = 6) are compared unstimulated sample median of the same experiment. Averages and ± SEM are indicated. *) p = 0.045; repeated measures ANOVA with Dunnett's multiple comparison test used.

observed in mice [56].

Early work with neutrophils showed that IL-4 enhanced neutrophil bactericidal activity [57] and that while IL-13 did not induce phagocytosis, it did induce neutrophil activation, RNA synthesis and IL-8-production [58]. A more recent mouse study found that signaling through type II IL-4R inhibits neutrophil migration and NET formation in bacterial and sterile inflammation [53]. Similar impaired NET formation and migration defect was also observed in neutrophils from allergic patients [59]. Overall, IL-4 signaling seems to protect against disease worsening in helminth infection [60] and joint inflammation [61,62] while it promotes resolution of inflammation in acute lung injury [63] or myocardial infarction [64]. It has been proposed that IL-4 and IL-13 serve as a mechanism to prevent tissue destruction caused by prolonged neutrophil action; as soon as type 2 effector cells take over and start producing IL-4 and IL-13 in tissues, neutrophils are "toned down" to avoid tissue damage [65–67].

We found that IL-4 and IL-13 induced several immune-related genes in highly purified human neutrophils. For example, clear induction of *IL-10* and simultaneous down-regulation of *TNF* appears logical for anti-inflammatory role of IL-4/IL-13 treated neutrophils. At the same time, IL-4/IL-13 both down-regulated expression of *LIF*, which in many instances is likely an anti-inflammatory cytokine; for example, in skeletal muscle regeneration, LIF provides an anti-inflammatory environment (reduced neutrophil influx and down-regulation of *IL-1b*, *IL-6* and *TNF*) in muscle regeneration phase after damage [68]. Also, LIF expression has been shown to have correlation with tumor-associated macrophages, and on the other hand, LIF inhibition/neutralization was able to induce tumor infiltration of CD8 + T cells, natural killer cells, and regulatory T cells [69].

*HRH4* was strongly up-regulated in neutrophils by IL-4 and IL-13, likely sensitizing them to histamine expression from mast cells albeit HRH4 may also negatively regulate neutrophil degranulation particularly when associated to cellular adhesion [70]. AnxA1 recruits monocytes and macrophages to clear apoptotic cells in the resolution phase of inflammation, which promotes the release of TGF-β and downplays proinflammatory IL-6 [71]. TGF-β signaling is also regulated by *CD109* that was clearly upregulated by IL-4 in neutrophils. CD109 is mostly expressed in activated T cells and platelets and its overexpression is connected with tumor progression, fibrosis and rheumatoid arthritis [72–74]. PTX3 is a soluble pattern recognition molecule that is upregulated during inflammation. It is stored in neutrophil granules and can be released into neutrophil extracellular traps [75]. Interestingly, PTX3 is increased in asthmatic airways, and it seems to negatively regulate Th17 development and IL-17A responses [76,77]. It has been shown to attenuate neutrophil recruitment by binding P-selectin [78]. In this light, our results might suggest that one immunosuppressive pathway regulated by IL-4 or IL-13 in neutrophils could be via PTX3. *CD200R1* was up-regulated upon IL-4/13 stimulation. It is a receptor for CD200 and this CD200–CD200R1 axis is known as a negative regulator of immune responses, including Th2 functions. For instance, it has been shown that CD200R1 engagement inhibits activation, proliferation and type 2 cytokine production on type 2 innate lymphoid cells (ILC2s) in allergic asthma [79] and deletion of *CD200R1* led in reduction of neutrophil ROS production as well as promotion of neutrophil niche in *Francisella tularensis* [80].

Intriguingly, we also discovered that IL-4 upregulates *PD-L2* RNA in neutrophils, which was confirmed by flow cytometry. The immunosuppressive PD-L2 has lately received attention as a target for checkpoint blockade therapy in cancer [81], making this finding particularly interesting. In our flow cytometry experiments we analyzed only viable neutrophil population for PD-L2 expression, but it is important to note, that after 20 h of incubation dead neutrophils were present in the culture, comprising a total of 15–30 % of all cells, which may cause bystander effects and needs further attention. The per cent of dead cells in the culture appeared more donor than stimulation dependent. This would imply that even if the PD-L2 expression was positively affected by the presence of dying cells in the culture, IL-4 did have an enhancing effect on it. Also, as the Histopaque-based neutrophil isolation method results in neutrophil purity that is lower than sorted neutrophil purity, it cannot be fully excluded that some contaminating mononuclear cells respond to the cytokine stimulus and secrete factors that, in turn, affect neutrophil PD-L2 expression.

During the immune response, cellular energy consumption increases. Neutrophils are thought to have only few mitochondria per cell and thus to use mainly glycolysis rather than mitochondrial oxidative phosphorylation as their ATP source [82]. In our experiments, neutrophils seemed to use both glycolysis and oxidative phosphorylation as a source for ATP production (see ECAR curve in Fig. 5A after 2-DG and Rot-AA applications). This calls for further studies, since neutrophils have been thought to have little or completely absent oxidative phosphorylation [51]. Interestingly, upon neutrophil IL-4 stimulation, expression of a gene phosphogluconate dehydrogenase (*PGD*) was increased. Upon the uptake of glucose, either glycolysis or pentose phosphate pathway (PPP) is utilized. PGD catalyzes the third reaction in PPP which then might be induced due IL-4 stimulation [83]. Since the effect is IL-4-specific (and not IL-13), it may occur via type I IL-4 receptor and one signaling difference between type I and type II IL-4R is the activation of insulin receptor substrate 2 (IRS2) [84]. However, for the time being, IRS2 appears to be more important for aerobic, not anaerobic glycolysis [85]. In macrophages and dendritic cells, antihelminth response involving IL-4 is related to glucose utilization via oxidative phosphorylation and ATP formation ensuring energy needs for prolonged (i.e. slow) response while rapid antibacterial response involving tissue swelling and hypoxia is linked to glucose utilization via glycolysis and subsequent biosynthesis of ATP [50].

Better understanding of neutrophils in various physiological environments will assist also in predicting their behavior various clinical situations. Sheer number of neutrophils is a double-edged sword; neutropenia will expose to infections while neutrophilia may result in tissue damage. Finally, in addition to cytokine-mediated activation of neutrophils other activation routes such as Toll-like receptors (TLRs) are important for their activation; deeper understanding of logics and integration of these various pathways in cell activation is needed to completely understand these powerful modulators of inflammation.

## Funding

This work was financially supported by the Academy of Finland (IJ; Grants 25013080481 and 25013142041), the Competitive State Research Financing of the Expert Responsibility area of Fimlab Laboratories (IJ; Grant X51409), Tampere Tuberculosis Foundation (IJ), Sigrid Juselius Foundation (IJ), Finnish Medical Foundation (IJ), Tampere Children's Hospital Support Association (IJ), and City of Tampere Science Foundation (LK), Nordlab Laboratories (IJ; Grant X3710-KT0011) and Orion research foundation (TS). This work was supported by the Intramural Research Program of the Center for Cancer Research, National Cancer Institute (NCI), Cancer Innovation Laboratory (CIL) under grant No. 1ZIABC009283-36. The views expressed in this article are those of the authors and do not necessarily reflect the official policy or position of the Department of Health and Human Services, nor does mention of trade names, commercial products, or organizations imply endorsement by the United States Government.

### *CRediT authorship contribution statement*

**Laura Kummola:** Conceptualization, Investigation, Methodology, Writing – original draft, Writing – review & editing. **Tanja Salomaa:** Investigation, Methodology, Writing – original draft, Writing – review & editing. **Zsuzsanna Ortutay:** Investigation, Methodology, Writing – original draft. **Ram Savan:** Conceptualization, Methodology, Writing – original draft. **Howard A. Young:** Conceptualization, Resources, Supervision, Writing – original draft. **Ilkka S. Junttila:** Conceptualization,

Funding acquisition, Supervision, Writing – original draft, Writing – review & editing.

## Declaration of Competing Interest

The authors declare that they have no known competing financial interests or personal relationships that could have appeared to influence the work reported in this paper.

## Data availability

Data will be made available on request.

## Acknowledgements

The authors acknowledge the Tampere facility of Flow Cytometry for their service. We also thank the Medical Bioinformatics Centre of Turku Bioscience Centre for the sequencing data analysis. The Centre is supported by University of Turku, Åbo Akademi University, Biocenter Finland and Elixir-Finland. Timothy Myers at NIAID Genomic Technologies Section is thanked for invaluable help with initial genome-wide data analysis of neutrophils. Reija Autio at Tampere University is thanked for her assistance with statistical analyses. Dedicated to the memory of William E. Paul (1936-2015), who inspired and oversaw the first experiments of this work.

## Appendix A. Supplementary material

Supplementary data to this article can be found online at https://doi.org/10.1016/j.cyto.2023.156159.

## References

[1] H.K. Lehman, B.H. Segal, The role of neutrophils in host defense and disease, J. Allergy Clin. Immunol. 145 (2020) 1535–1544, https://doi.org/10.1016/j.jaci.2020.02.038.
[2] K. Ley, H.M. Hoffman, P. Kubes, M.A. Cassatella, A. Zychlinsky, C.C. Hedrick, S. D. Catz, Neutrophils: New insights and open questions, Sci. Immunol. 3 (2018) eaat4579, https://doi.org/10.1126/sciimmunol.aat4579.
[3] W. Weninger, M. Biro, R. Jain, Leukocyte migration in the interstitial space of non-lymphoid organs, Nat. Rev. Immunol. 14 (2014) 232–246, https://doi.org/10.1038/nri3641.
[4] S.J. Galli, N. Borregaard, T.A. Wynn, Phenotypic and functional plasticity of cells of innate immunity: macrophages, mast cells and neutrophils, Nat. Immunol. 12 (2011) 1035–1044, https://doi.org/10.1038/ni.2109.
[5] A. Mantovani, M.A. Cassatella, C. Costantini, S. Jaillon, Neutrophils in the activation and regulation of innate and adaptive immunity, Nat. Rev. Immunol. 11 (2011) 519–531, https://doi.org/10.1038/nri3024.
[6] P. Scapini, O. Marini, C. Tecchio, M.A. Cassatella, Human neutrophils in the saga of cellular heterogeneity: insights and open questions, Immunol. Rev. 273 (2016) 48–60, https://doi.org/10.1111/imr.12448.
[7] L.A. González, F. Melo-González, V.P. Sebastián, O.P. Vallejos, L.P. Noguera, I. D. Suazo, B.M. Schultz, A.H. Manosalva, H.F. Peñaloza, J.A. Soto, D. Parker, C. A. Riedel, P.A. González, A.M. Kalergis, S.M. Bueno, Characterization of the Anti-Inflammatory Capacity of IL-10-Producing Neutrophils in Response to Streptococcus pneumoniae Infection, Front. Immunol. 12 (2021), 638917, https://doi.org/10.3389/fimmu.2021.638917.
[8] X. Zhang, L. Majlessi, E. Deriaud, C. Leclerc, R. Lo-Man, Coactivation of Syk kinase and MyD88 adaptor protein pathways by bacteria promotes regulatory properties of neutrophils, Immunity. 31 (2009) 761–771, https://doi.org/10.1016/j.immuni.2009.09.016.
[9] N.L. Bowers, E.S. Helton, R.P.H. Huijbregts, P.A. Goepfert, S.L. Heath, Z. Hel, Immune suppression by neutrophils in HIV-1 infection: role of PD-L1/PD-1 pathway, PLoS Pathog. 10 (2014) e1003993.
[10] S. de Kleijn, J.D. Langereis, J. Leentjens, M. Kox, M.G. Netea, L. Koenderman, G. Ferwerda, P. Pickkers, P.W.M. Hermans, IFN-γ-stimulated neutrophils suppress lymphocyte proliferation through expression of PD-L1, PloS One. 8 (2013) e72249.
[11] G. He, H. Zhang, J. Zhou, B. Wang, Y. Chen, Y. Kong, X. Xie, X. Wang, R. Fei, L. Wei, H. Chen, H. Zeng, Peritumoural neutrophils negatively regulate adaptive immunity via the PD-L1/PD-1 signalling pathway in hepatocellular carcinoma, J. Exp. Clin. Cancer Res. CR. 34 (2015) 141, https://doi.org/10.1186/s13046-015-0256-0.
[12] Y. Pico de Coaña, I. Poschke, G. Gentilcore, Y. Mao, M. Nyström, J. Hansson, G. V. Masucci, R. Kiessling, Ipilimumab treatment results in an early decrease in the frequency of circulating granulocytic myeloid-derived suppressor cells as well as their Arginase1 production, Cancer, Immunol. Res. 1 (2013) 158–162, https://doi.org/10.1158/2326-6066.CIR-13-0016.
[13] R. Rotondo, G. Barisione, L. Mastracci, F. Grossi, A.M. Orengo, R. Costa, M. Truini, M. Fabbi, S. Ferrini, O. Barbieri, IL-8 induces exocytosis of arginase 1 by neutrophil polymorphonuclears in nonsmall cell lung cancer, Int. J. Cancer. 125 (2009) 887–893, https://doi.org/10.1002/ijc.24448.
[14] A. Breedveld, T. Groot Kormelink, M. van Egmond, E.C. de Jong, Granulocytes as modulators of dendritic cell function, J. Leukoc. Biol. 102 (2017) 1003–1016, https://doi.org/10.1189/jlb.4MR0217-048RR.
[15] T.M. Finlay, A.L. Palmer, S.S. Ousman, Murine neutrophils treated with alphaB-crystallin reduce IL-12p40 production by dendritic cells, Immunology. 155 (2018) 72–84, https://doi.org/10.1111/imm.12924.
[16] K. Moses, S. Brandau, Human neutrophils: Their role in cancer and relation to myeloid-derived suppressor cells, Semin. Immunol. 28 (2016) 187–196, https://doi.org/10.1016/j.smim.2016.03.018.
[17] E. Uribe-Querol, C. Rosales, Neutrophils in Cancer: Two Sides of the Same Coin, J. Immunol. Res. 2015 (2015), 983698, https://doi.org/10.1155/2015/983698.
[18] A. De Benedetto, R. Agnihothri, L.Y. McGirt, L.G. Bankova, L.A. Beck, Atopic dermatitis: a disease caused by innate immune defects? J. Invest. Dermatol. 129 (2009) 14–30, https://doi.org/10.1038/jid.2008.259.
[19] A. Ray, M. Raundhal, T.B. Oriss, P. Ray, S.E. Wenzel, Current concepts of severe asthma, J. Clin. Invest. 126 (2016) 2394–2403, https://doi.org/10.1172/JCI84144.
[20] A. Ray, J.K. Kolls, Neutrophilic Inflammation in Asthma and Association with Disease Severity, Trends Immunol. 38 (2017) 942–954, https://doi.org/10.1016/j.it.2017.07.003.
[21] D. Polak, C. Hafner, P. Briza, C. Kitzmüller, A. Elbe-Bürger, N. Samadi, M. Gschwandtner, W. Pfützner, G.J. Zlabinger, B. Jahn-Schmid, B. Bohle, A novel role for neutrophils in IgE-mediated allergy: Evidence for antigen presentation in late-phase reactions, J. Allergy Clin. Immunol. 143 (2019) 1143–1152.e4, https://doi.org/10.1016/j.jaci.2018.06.005.
[22] S. Bonne-Année, L.A. Kerepesi, J.A. Hess, A.E. O'Connell, J.B. Lok, T.J. Nolan, D. Abraham, Human and mouse macrophages collaborate with neutrophils to kill larval Strongyloides stercoralis, Infect. Immun. 81 (2013) 3346–3355, https://doi.org/10.1128/IAI.00625-13.
[23] S. Bonne-Année, L.A. Kerepesi, J.A. Hess, J. Wesolowski, F. Paumet, J.B. Lok, T. J. Nolan, D. Abraham, Extracellular traps are associated with human and mouse neutrophil and macrophage mediated killing of larval Strongyloides stercoralis, Microbes Infect. 16 (2014) 502–511, https://doi.org/10.1016/j.micinf.2014.02.012.
[24] T.E. Sutherland, N. Logan, D. Rückerl, A.A. Humbles, S.M. Allan, V. Papayannopoulos, B. Stockinger, R.M. Maizels, J.E. Allen, Chitinase-like proteins promote IL-17-mediated neutrophilia in a tradeoff between nematode killing and host damage, Nat. Immunol. 15 (2014) 1116–1125, https://doi.org/10.1038/ni.3023.
[25] F. Chen, W. Wu, A. Millman, J.F. Craft, E. Chen, N. Patel, J.L. Boucher, J.F. Urban, C.C. Kim, W.C. Gause, Neutrophils prime a long-lived effector macrophage phenotype that mediates accelerated helminth expulsion, Nat. Immunol. 15 (2014) 938–946, https://doi.org/10.1038/ni.2984.
[26] C. Radermecker, R. Louis, F. Bureau, T. Marichal, Role of neutrophils in allergic asthma, Curr. Opin. Immunol. 54 (2018) 28–34, https://doi.org/10.1016/j.coi.2018.05.006.
[27] T.A. Wynn, Type 2 cytokines: mechanisms and therapeutic strategies, Nat. Rev. Immunol. 15 (2015) 271–282, https://doi.org/10.1038/nri3831.
[28] J. Zhu, T helper 2 (Th2) cell differentiation, type 2 innate lymphoid cell (ILC2) development and regulation of interleukin-4 (IL-4) and IL-13 production, Cytokine. 75 (2015) 14–24, https://doi.org/10.1016/j.cyto.2015.05.010.
[29] I.S. Junttila, Tuning the Cytokine Responses: An Update on Interleukin (IL)-4 and IL-13 Receptor Complexes, Front. Immunol. 9 (2018) 888, https://doi.org/10.3389/fimmu.2018.00888.
[30] D.B. Kuhns, D.A.L. Priel, J. Chu, K.A. Zarember, Isolation and Functional Analysis of Human Neutrophils, Curr. Protoc. Immunol. 111 (2015) 7.23.1-7.23.16. 10.1002/0471142735.im0723s111.
[31] D.A. Dorward, C.D. Lucas, A.L. Alessandri, J.A. Marwick, F. Rossi, I. Dransfield, C. Haslett, K. Dhaliwal, A.G. Rossi, Technical advance: autofluorescence-based sorting: rapid and nonperturbing isolation of ultrapure neutrophils to determine cytokine production, J. Leukoc. Biol. 94 (2013) 193–202, https://doi.org/10.1189/jlb.0113040.
[32] G. de Sena Brandine, A.D. Smith, Falco: high-speed FastQC emulation for quality control of sequencing data, F1000Research. 8 (2019) 1874. 10.12688/f1000research.21142.2.
[33] P. Ewels, M. Magnusson, S. Lundin, M. Käller, MultiQC: summarize analysis results for multiple tools and samples in a single report, Bioinforma. Oxf. Engl. 32 (2016) 3047–3048, https://doi.org/10.1093/bioinformatics/btw354.
[34] Y. Liao, G.K. Smyth, W. Shi, The R package Rsubread is easier, faster, cheaper and better for alignment and quantification of RNA sequencing reads, Nucleic Acids Res. 47 (2019) e47.
[35] M.I. Love, W. Huber, S. Anders, Moderated estimation of fold change and dispersion for RNA-seq data with DESeq2, Genome Biol. 15 (2014) 550, https://doi.org/10.1186/s13059-014-0550-8.
[36] M.I. Love, C. Ahlmann-Eltze, K. Forbes, S. Anders, W. Huber, Bioconductor, (n.d.). https://bioconductor.org/packages/release/bioc/html/DESeq2.html (accessed November 28, 2022).
[37] E.Y. Chen, C.M. Tan, Y. Kou, Q. Duan, Z. Wang, G.V. Meirelles, N.R. Clark, A. Ma'ayan, Enrichr: interactive and collaborative HTML5 gene list enrichment analysis tool, BMC Bioinformatics. 14 (2013) 128. 10.1186/1471-2105-14-128.

[38] M.V. Kuleshov, M.R. Jones, A.D. Rouillard, N.F. Fernandez, Q. Duan, Z. Wang, S. Koplev, S.L. Jenkins, K.M. Jagodnik, A. Lachmann, M.G. McDermott, C.D. Monteiro, G.W. Gundersen, A. Ma'ayan, Enrichr: a comprehensive gene set enrichment analysis web server 2016 update, Nucleic Acids Res. 44 (2016) W90-97. 10.1093/nar/gkw377.
[39] A.L. Tarca, S. Draghici, P. Khatri, S.S. Hassan, P. Mittal, J.-S. Kim, C.J. Kim, J. P. Kusanovic, R. Romero, A novel signaling pathway impact analysis, Bioinforma. Oxf. Engl. 25 (2009) 75–82, https://doi.org/10.1093/bioinformatics/btn577.
[40] A.L. Tarca, P. Kathri, S. Draghici, Bioconductor, (n.d.). https://bioconductor.org/packages/release/bioc/html/SPIA.html (accessed November 28, 2022).
[41] A. Zhu, J.G. Ibrahim, M.I. Love, Heavy-tailed prior distributions for sequence count data: removing the noise and preserving large differences, Bioinforma. Oxf. Engl. 35 (2019) 2084–2092, https://doi.org/10.1093/bioinformatics/bty895.
[42] R. Kolde, Pheatmap: Pretty Heatmaps, (n.d.). https://cran.r-project.org/web/packages/pheatmap/index.html (accessed November 28, 2022).
[43] R. Savan, A.P. McFarland, D.A. Reynolds, L. Feigenbaum, K. Ramakrishnan, M. Karwan, H. Shirota, D.M. Klinman, K. Dunleavy, S. Pittaluga, S.K. Anderson, R. P. Donnelly, W.H. Wilson, H.A. Young, A novel role for IL-22R1 as a driver of inflammation, Blood. 117 (2011) 575–584, https://doi.org/10.1182/blood-2010-05-285908.
[44] C. Solinas, M. Aiello, E. Rozali, M. Lambertini, K. Willard-Gallo, E. Migliori, Programmed cell death-ligand 2: A neglected but important target in the immune response to cancer? Transl. Oncol. 13 (2020), 100811 https://doi.org/10.1016/j.tranon.2020.100811.
[45] T. Yamazaki, H. Akiba, H. Iwai, H. Matsuda, M. Aoki, Y. Tanno, T. Shin, H. Tsuchiya, D.M. Pardoll, K. Okumura, M. Azuma, H. Yagita, Expression of programmed death 1 ligands by murine T cells and APC, J. Immunol. Baltim. Md 1950 (169) (2002) 5538–5545, https://doi.org/10.4049/jimmunol.169.10.5538.
[46] K. Bratke, L. Fritz, F. Nokodian, K. Geißler, K. Garbe, M. Lommatzsch, J.C. Virchow, Differential regulation of PD-1 and its ligands in allergic asthma, Clin. Exp. Allergy J. Br. Soc. Allergy Clin. Immunol. 47 (2017) 1417–1425, https://doi.org/10.1111/cea.13017.
[47] A. Ivetic, H.L. Hoskins Green, S.J. Hart, L-selectin: A Major Regulator of Leukocyte Adhesion, Migration and Signaling, Front. Immunol. 10 (2019) 1068, https://doi.org/10.3389/fimmu.2019.01068.
[48] S. Mol, F.M.J. Hafkamp, L. Varela, N. Simkhada, E.W. Taanman-Kueter, S.W. Tas, M.H.M. Wauben, T. Groot Kormelink, E.C. de Jong, Efficient Neutrophil Activation Requires Two Simultaneous Activating Stimuli, Int. J. Mol. Sci. 22 (2021) 10106, https://doi.org/10.3390/ijms221810106.
[49] J. Pillay, V.M. Kamp, E. van Hoffen, T. Visser, T. Tak, J.-W. Lammers, L.H. Ulfman, L.P. Leenen, P. Pickkers, L. Koenderman, A subset of neutrophils in human systemic inflammation inhibits T cell responses through Mac-1, J. Clin. Invest. 122 (2012) 327–336, https://doi.org/10.1172/JCI57990.
[50] L.A.J. O'Neill, R.J. Kishton, J. Rathmell, A guide to immunometabolism for immunologists, Nat. Rev. Immunol. 16 (2016) 553–565, https://doi.org/10.1038/nri.2016.70.
[51] P.A. Kramer, S. Ravi, B. Chacko, M.S. Johnson, V.M. Darley-Usmar, A review of the mitochondrial and glycolytic metabolism in human platelets and leukocytes: implications for their use as bioenergetic biomarkers, Redox Biol. 2 (2014) 206–210, https://doi.org/10.1016/j.redox.2013.12.026.
[52] J. Lahoz-Beneytez, M. Elemans, Y. Zhang, R. Ahmed, A. Salam, M. Block, C. Niederalt, B. Asquith, D. Macallan, Human neutrophil kinetics: modeling of stable isotope labeling data supports short blood neutrophil half-lives, Blood. 127 (2016) 3431–3438, https://doi.org/10.1182/blood-2016-03-700336.
[53] J. Woytschak, N. Keller, C. Krieg, D. Impellizzieri, R.W. Thompson, T.A. Wynn, A. S. Zinkernagel, O. Boyman, Type 2 Interleukin-4 Receptor Signaling in Neutrophils Antagonizes Their Expansion and Migration during Infection and Inflammation, Immunity. 45 (2016) 172–184, https://doi.org/10.1016/j.immuni.2016.06.025.
[54] I.S. Junttila, K. Mizukami, H. Dickensheets, M. Meier-Schellersheim, H. Yamane, R. P. Donnelly, W.E. Paul, Tuning sensitivity to IL-4 and IL-13: differential expression of IL-4Ralpha, IL-13Ralpha1, and gammac regulates relative cytokine sensitivity, J. Exp. Med. 205 (2008) 2595–2608, https://doi.org/10.1084/jem.20080452.
[55] J.A. Sosman, S.G. Fisher, C. Kefer, R.I. Fisher, T.M. Ellis, A phase I trial of continuous infusion interleukin-4 (IL-4) alone and following interleukin-2 (IL-2) in cancer patients, Ann. Oncol. Off. J. Eur. Soc. Med. Oncol. 5 (1994) 447–452, https://doi.org/10.1093/oxfordjournals.annonc.a058878.
[56] J.D. Milner, T. Orekov, J.M. Ward, L. Cheng, F. Torres-Velez, I. Junttila, G. Sun, M. Buller, S.C. Morris, F.D. Finkelman, W.E. Paul, Sustained IL-4 exposure leads to a novel pathway for hemophagocytosis, inflammation, and tissue macrophage accumulation, Blood. 116 (2010) 2476–2483, https://doi.org/10.1182/blood-2009-11-255174.
[57] H. Boey, R. Rosenbaum, J. Castracane, L. Borish, Interleukin-4 is a neutrophil activator, J. Allergy Clin. Immunol. 83 (1989) 978–984, https://doi.org/10.1016/0091-6749(89)90115-2.
[58] D. Girard, R. Paquin, P.H. Naccache, A.D. Beaulieu, Effects of interleukin-13 on human neutrophil functions, J. Leukoc. Biol. 59 (1996) 412–419, https://doi.org/10.1002/jlb.59.3.412.
[59] D. Impellizzieri, F. Ridder, M.E. Raeber, C. Egholm, J. Woytschak, A.G.A. Kolios, D. F. Legler, O. Boyman, IL-4 receptor engagement in human neutrophils impairs their migration and extracellular trap formation, J. Allergy Clin. Immunol. 144 (2019) 267–279.e4, https://doi.org/10.1016/j.jaci.2019.01.042.
[60] F. Chen, Z. Liu, W. Wu, C. Rozo, S. Bowdridge, A. Millman, N. Van Rooijen, J. F. Urban, T.A. Wynn, W.C. Gause, An essential role for TH2-type responses in limiting acute tissue damage during experimental helminth infection, Nat. Med. 18 (2012) 260–266, https://doi.org/10.1038/nm.2628.
[61] S.K. Panda, G. Wigerblad, L. Jiang, Y. Jiménez-Andrade, V.S. Iyer, Y. Shen, S. V. Boddul, A.O. Guerreiro-Cacais, B. Raposo, Z. Kasza, F. Wermeling, IL-4 controls activated neutrophil FcγR2b expression and migration into inflamed joints, Proc. Natl. Acad. Sci. U. S. A. 117 (2020) 3103–3113, https://doi.org/10.1073/pnas.1914186117.
[62] A.S. Schmid, T. Hemmerle, F. Pretto, A. Kipar, D. Neri, Antibody-based targeted delivery of interleukin-4 synergizes with dexamethasone for the reduction of inflammation in arthritis, Rheumatol. Oxf. Engl. 57 (2018) 748–755, https://doi.org/10.1093/rheumatology/kex447.
[63] A.J. Harris, A.S. Mirchandani, R.W. Lynch, F. Murphy, L. Delaney, D. Small, P. Coelho, E.R. Watts, P. Sadiku, D. Griffith, R.S. Dickinson, E. Clark, J.A. Willson, T. Morrison, M. Mazzone, P. Carmeliet, B. Ghesquiere, C. O'Kane, D. McAuley, S. J. Jenkins, M.K.B. Whyte, S.R. Walmsley, IL4Rα Signaling Abrogates Hypoxic Neutrophil Survival and Limits Acute Lung Injury Responses In Vivo, Am. J. Respir. Crit. Care Med. 200 (2019) 235–246, https://doi.org/10.1164/rccm.201808-1599OC.
[64] M.J. Daseke, M.A.A. Tenkorang-Impraim, Y. Ma, U. Chalise, S.R. Konfrst, M. R. Garrett, K.Y. DeLeon-Pennell, M.L. Lindsey, Exogenous IL-4 shuts off pro-inflammation in neutrophils while stimulating anti-inflammation in macrophages to induce neutrophil phagocytosis following myocardial infarction, J. Mol. Cell. Cardiol. 145 (2020) 112–121, https://doi.org/10.1016/j.yjmcc.2020.06.006.
[65] C. Egholm, L.E.M. Heeb, D. Impellizzieri, O. Boyman, The Regulatory Effects of Interleukin-4 Receptor Signaling on Neutrophils in Type 2 Immune Responses, Front. Immunol. 10 (2019) 2507, https://doi.org/10.3389/fimmu.2019.02507.
[66] L.E.M. Heeb, C. Egholm, D. Impellizzieri, F. Ridder, O. Boyman, Regulation of neutrophils in type 2 immune responses, Curr. Opin. Immunol. 54 (2018) 115–122, https://doi.org/10.1016/j.coi.2018.06.009.
[67] L.E.M. Heeb, C. Egholm, O. Boyman, Evolution and function of interleukin-4 receptor signaling in adaptive immunity and neutrophils, Genes Immun. 21 (2020) 143–149, https://doi.org/10.1038/s41435-020-0095-7.
[68] L.C. Hunt, A. Upadhyay, J.A. Jazayeri, E.M. Tudor, J.D. White, An anti-inflammatory role for leukemia inhibitory factor receptor signaling in regenerating skeletal muscle, Histochem. Cell Biol. 139 (2013) 13–34, https://doi.org/10.1007/s00418-012-1018-0.
[69] M. Pascual-García, E. Bonfill-Teixidor, E. Planas-Rigol, C. Rubio-Perez, R. Iurlaro, A. Arias, I. Cuartas, A. Sala-Hojman, L. Escudero, F. Martínez-Ricarte, I. Huber-Ruano, P. Nuciforo, L. Pedrosa, C. Marques, I. Braña, E. Garralda, M. Vieito, M. Squatrito, E. Pineda, F. Graus, C. Espejo, J. Sahuquillo, J. Tabernero, J. Seoane, LIF regulates CXCL9 in tumor-associated macrophages and prevents CD8+ T cell tumor-infiltration impairing anti-PD1 therapy, Nat. Commun. 10 (2019) 2416, https://doi.org/10.1038/s41467-019-10369-9.
[70] K. Dib, T. Perecko, V. Jenei, C. McFarlane, D. Comer, V. Brown, M. Katebe, T. Scheithauer, R.L. Thurmond, P.L. Chazot, M. Ennis, The histamine H4 receptor is a potent inhibitor of adhesion-dependent degranulation in human neutrophils, J. Leukoc. Biol. 96 (2014) 411–418, https://doi.org/10.1189/jlb.2AB0813-432RR.
[71] M.A. Sugimoto, J.P. Vago, M.M. Teixeira, L.P. Sousa, Annexin A1 and the Resolution of Inflammation: Modulation of Neutrophil Recruitment, Apoptosis, and Clearance, J. Immunol. Res. 2016 (2016) 8239258, https://doi.org/10.1155/2016/8239258.
[72] A.A. Bizet, N. Tran-Khanh, A. Saksena, K. Liu, M.D. Buschmann, A. Philip, CD109-mediated degradation of TGF-β receptors and inhibition of TGF-β responses involve regulation of SMAD7 and Smurf2 localization and function, J. Cell. Biochem. 113 (2012) 238–246, https://doi.org/10.1002/jcb.23349.
[73] S. Hagiwara, Y. Murakumo, S. Mii, T. Shigetomi, N. Yamamoto, H. Furue, M. Ueda, M. Takahashi, Processing of CD109 by furin and its role in the regulation of TGF-beta signaling, Oncogene. 29 (2010) 2181–2191, https://doi.org/10.1038/onc.2009.506.
[74] G. Song, T. Feng, R. Zhao, Q. Lu, Y. Diao, Q. Guo, Z. Wang, Y. Zhang, L. Ge, J. Pan, L. Wang, J. Han, CD109 regulates the inflammatory response and is required for the pathogenesis of rheumatoid arthritis, Ann. Rheum. Dis. 78 (2019) 1632–1641, https://doi.org/10.1136/annrheumdis-2019-215473.
[75] S. Jaillon, G. Peri, Y. Delneste, I. Frémaux, A. Doni, F. Moalli, C. Garlanda, L. Romani, H. Gascan, S. Bellocchio, S. Bozza, M.A. Cassatella, P. Jeannin, A. Mantovani, The humoral pattern recognition receptor PTX3 is stored in neutrophil granules and localizes in extracellular traps, J. Exp. Med. 204 (2007) 793–804, https://doi.org/10.1084/jem.20061301.
[76] J. Balhara, L. Shan, J. Zhang, A. Muhuri, A.J. Halayko, M.S. Almiski, D. Doeing, J. McConville, M.M. Matzuk, A.S. Gounni, Pentraxin 3 deletion aggravates allergic inflammation through a TH17-dominant phenotype and enhanced CD4 T-cell survival, J. Allergy Clin. Immunol. 139 (2017) 950–963.e9, https://doi.org/10.1016/j.jaci.2016.04.063.
[77] G. Gupta, Z. Mou, P. Jia, R. Sharma, R. Zayats, S.M. Viana, L. Shan, A. Barral, V. S. Boaventura, T.T. Murooka, A. Soussi-Gounni, C.I. de Oliveira, J.E. Uzonna, The Long Pentraxin 3 (PTX3) Suppresses Immunity to Cutaneous Leishmaniasis by Regulating CD4+ T Helper Cell Response, Cell Rep. 33 (2020), 108513, https://doi.org/10.1016/j.celrep.2020.108513.
[78] L. Deban, R.C. Russo, M. Sironi, F. Moalli, M. Scanziani, V. Zambelli, I. Cuccovillo, A. Bastone, M. Gobbi, S. Valentino, A. Doni, C. Garlanda, S. Danese, G. Salvatori, M. Sassano, V. Evangelista, B. Rossi, E. Zenaro, G. Constantin, C. Laudanna, B. Bottazzi, A. Mantovani, Regulation of leukocyte recruitment by the long pentraxin PTX3, Nat. Immunol. 11 (2010) 328–334, https://doi.org/10.1038/ni.1854.
[79] P. Shafiei-Jahani, D.G. Helou, B.P. Hurrell, E. Howard, C. Quach, J.D. Painter, L. Galle-Treger, M. Li, Y.-H.-E. Loh, O. Akbari, CD200-CD200R immune checkpoint engagement regulates ILC2 effector function and ameliorates lung inflammation in

asthma, Nat. Commun. 12 (2021) 2526, https://doi.org/10.1038/s41467-021-22832-7.
[80] J. Casulli, M.E. Fife, S.A. Houston, S. Rossi, J. Dow, E.D. Williamson, G.C. Clark, T. Hussell, R.V. D'Elia, M.A. Travis, CD200R deletion promotes a neutrophil niche for Francisella tularensis and increases infectious burden and mortality, Nat. Commun. 10 (2019) 2121, https://doi.org/10.1038/s41467-019-10156-6.
[81] Y. Wang, J. Du, Z. Gao, H. Sun, M. Mei, Y. Wang, Y. Ren, X. Zhou, Evolving landscape of PD-L2: bring new light to checkpoint immunotherapy, Br. J. Cancer. (2022), https://doi.org/10.1038/s41416-022-02084-y.
[82] M. Na, G. J, S. Sm, A. Es, R. D, K. Tw, Functional characterization of mitochondria in neutrophils: a role restricted to apoptosis, Cell Death Differ. 11 (2004). 10.1038/sj.cdd.4401320.
[83] S. Daneshmandi, T. Cassel, R.M. Higashi, T.-W.-M. Fan, P. Seth, 6-Phosphogluconate dehydrogenase (6PGD), a key checkpoint in reprogramming of regulatory T cells metabolism and function, ELife. 10 (2021) e67476.
[84] N.M. Heller, X. Qi, I.S. Junttila, K.A. Shirey, S.N. Vogel, W.E. Paul, A.D. Keegan, Type I IL-4Rs selectively activate IRS-2 to induce target gene expression in macrophages, Sci. Signal. 1 (2008) ra17, https://doi.org/10.1126/scisignal.1164795.
[85] J. Landis, L.M. Shaw, Insulin receptor substrate 2-mediated phosphatidylinositol 3-kinase signaling selectively inhibits glycogen synthase kinase 3β to regulate aerobic glycolysis, J. Biol. Chem. 289 (2014) 18603–18613, https://doi.org/10.1074/jbc.M114.564070.

# Supplementary Figure 1

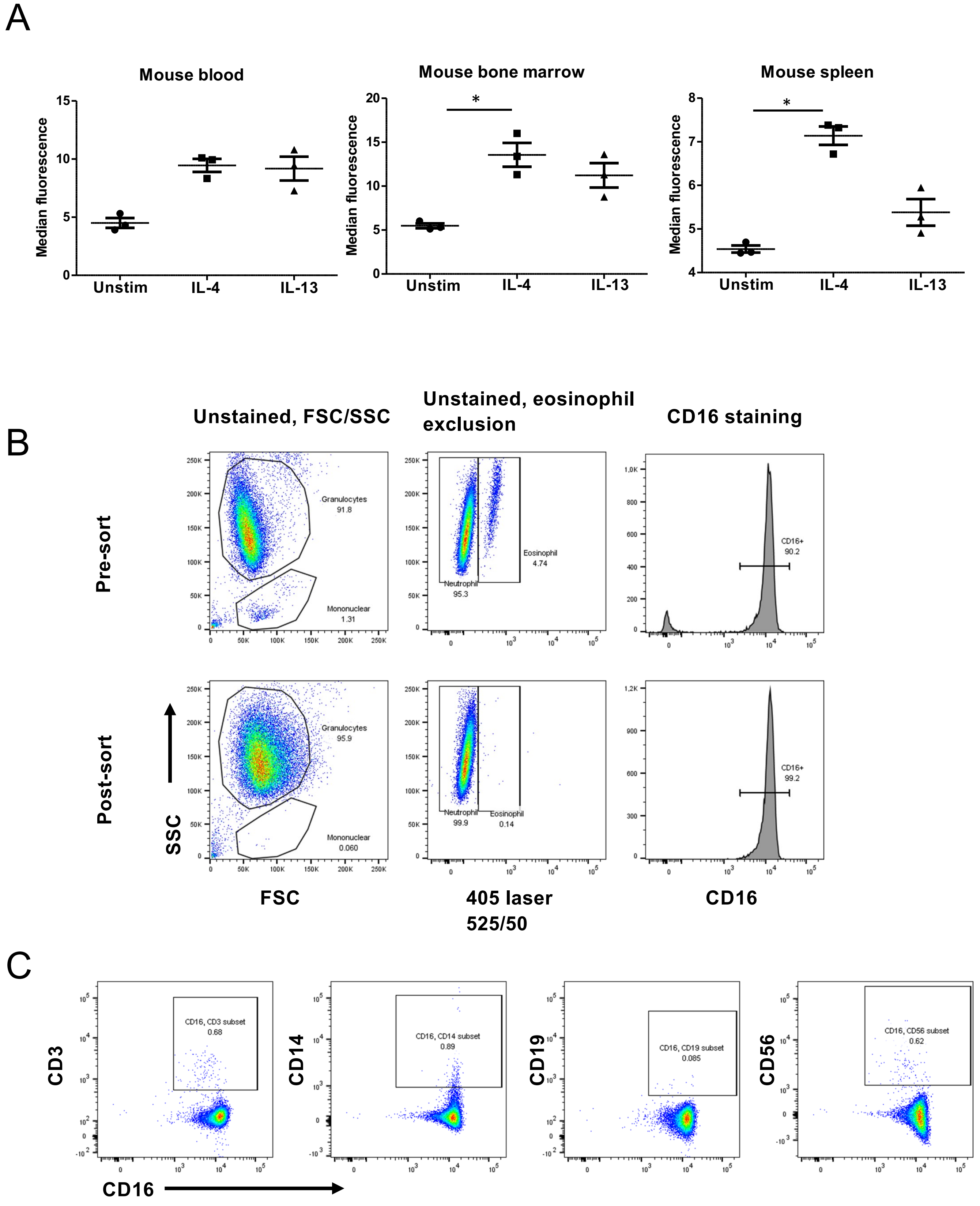


Supplementary Figure 1. (A) Blood, bone marrow and splenic primary neutrophils of three *IL-22R1* transgenic mice were enriched and stimulated with IL-4 or IL-13 for 15 minutes. Median fluorescence intensities for pSTAT6 in Gr-1$^{high}$ cells are shown. *) p =0.0429, for bone marrow and spleen Friedman test with Dunn's multiple comparisons test. (B) Human neutrophils were sorted based on forward and side scatter characteristics and low autofluorescence. Plots during and after sort are shown, along with CD16 staining of pre- and post-sorted cells. (C) After sorting, cells were stained for CD3, CD14, CD16, CD19 and CD56.

# Supplementary Figure 2

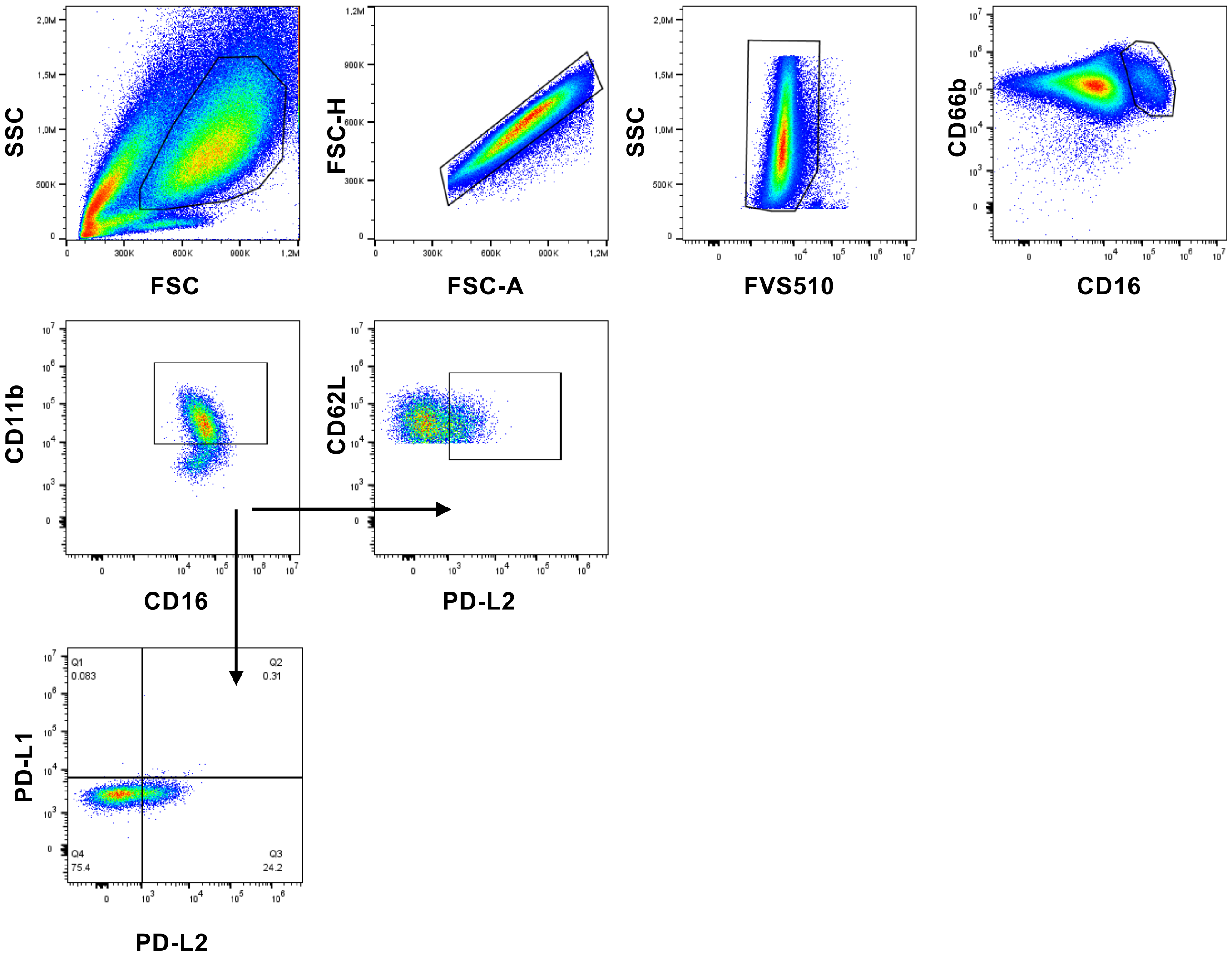


Supplementary Figure 2. Gating strategy for PD-L2 staining in human IL-4 and IL-13 stimulated neutrophils (20 hours, 100 ng/ml). Gating proceeds hierarchically from left to right or as indicated by arrows.

Supplementary Figure 3

A

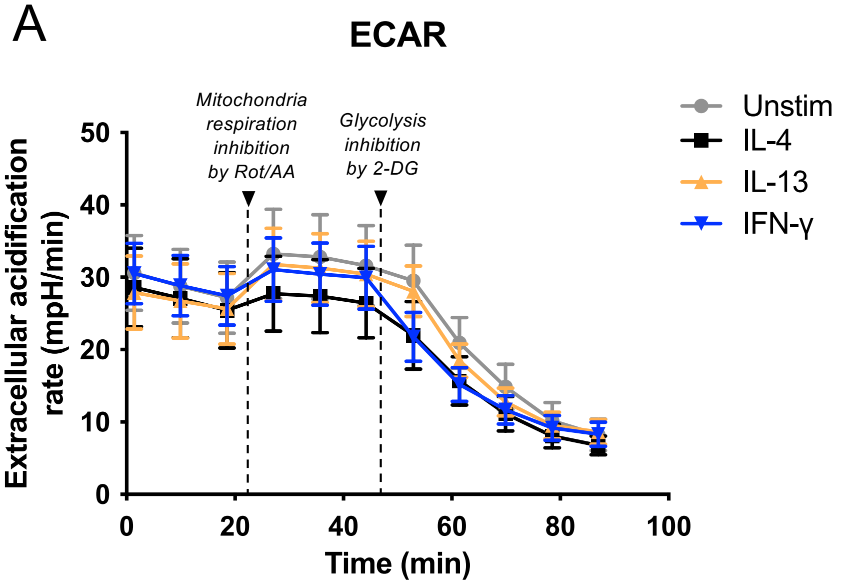


B

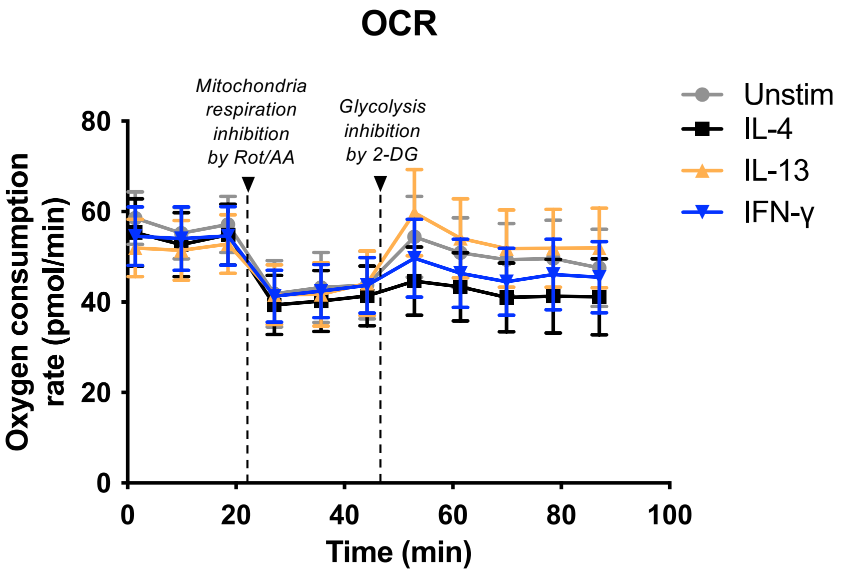


Supplementary Figure 3. (A) Extracellular acidification (ECAR) and (B) oxygen consumption rate (OCR) were measured in milli-pH per minute (mpH/min) for ECAR and pmol/min for OCR for 11-cycles. After measuring 3 basal level measurements, 3 measurements were recorded to determine the effect of Rotenone and Antimycin A (Rot/AA) for inhibiting mitochondrial respiration. Additional 5 measurements after injection of 2-deoxyglucose (2-DG) for glycolysis inhibition were detected. Measurement interval time was 8 min 30 sec. Averages and ±SEM of the medians of each experiment (n=6) in each condition (unstimulated or stimulation with IL-4, IL-13 or I IFN-γ 50 ng/ml for 1 hour) are shown.

| **Analysis/Antibody** | **Fluorochrome** | **Clone** | **Vendor** |
|---|---|---|---|
| **Human phospho-STAT** | | | |
| anti-CD11b | FITC | ICRF44 | eBioscience |
| anti-CD16 | APC | eBioCB16 | eBioscience |
| anti-pSTAT1 | PE | 4a | BD |
| anti-pSTAT6 | PE | 18/P-Stat6 | BD |
| | | | |
| **Sorting purity** | | | |
| anti-CD3 | APC | UCHT1 | BD |
| anti-CD14 | FITC | M5E2 | BD |
| anti-CD16 | APC-Cy7 | 3G8 | BD |
| anti-CD19 | PerCP-Cy5.5 | HIB19 | BD |
| anti-CD56 | PE-Cy7 | B159 | BD |
| | | | |
| **PD-L1 and PD-L2 expression** | | | |
| anti-PD-L1 (CD274) | BV421 | MIH3 | BioLegend |
| anti-PD-L2 (CD273) | BV650 | EH12.2H7 | BioLegend |
| anti-CD11b | APC | ICRF44 | eBioscience |
| anti-CD16 | APC-Cy7 | 3G8 | BD |
| anti-CD66b | BB515 | G10F5 | BD |
| anti-CD62L | PerCP-Cy5.5 | DREG.56 | eBioscience |
| Fixable Viability Stain | FVS510 | n/a | BD |
| | | | |
| **Mouse phospho-STAT** | | | |
| anti-pSTAT6 | AlexaFluor 647 | J71-773.58.11 | BD |
| anti-Gr1 | FITC | 1A8 | BD |

Supplementary Table 1. Flow cytometry antibody and panel details.

## Supplementary table 2

| | | IL-4 stimulation | |
|---|---|---|---|
| **Entrez ID** | **Symbol** | **log2foldchange** | **padj** |
| 59340 | HRH4 | 8,296076321 | 1,33548E-10 |
| 1154 | CISH | 7,589738416 | 3,00262E-57 |
| 100506810 | LINC01132 | 7,431306195 | 5,29894E-09 |
| 131450 | CD200R1 | 7,101566028 | 1,51941E-17 |
| 8651 | SOCS1 | 6,241303754 | 3,03252E-46 |
| 445347 | TARP | 6,120951863 | 4,27935E-12 |
| 80380 | PDCD1LG2 | 6,368431955 | 5,32946E-05 |
| 84848 | MIR503HG | | |
| 162073 | ITPRIPL2 | 5,515894849 | 1,17532E-17 |
| 5473 | PPBP | 5,138780126 | 0,000353585 |
| 81029 | WNT5B | 5,31623333 | 0,000932398 |
| 283237 | TTC9C | 5,292372381 | 3,13244E-55 |
| 3586 | IL10 | 5,446333883 | 0,0001017 |
| 5028 | P2RY1 | 5,441444684 | 0,000507534 |
| 6967 | TRGC2 | 4,915142308 | 1,63148E-06 |
| 4628 | MYH10 | 4,814113929 | 0,003268305 |
| 3321 | IGSF3 | 5,032388152 | 1,40019E-18 |
| 653075 | GOLGA8T | 4,198708203 | 0,042094461 |
| 144100 | PLEKHA7 | 4,664240586 | 0,01301415 |
| 8793 | TNFRSF10D | 4,64245964 | 5,02807E-33 |
| 64805 | P2RY12 | 4,538434919 | 0,022671589 |
| 387837 | CLEC12B | 4,495223778 | 1,38278E-15 |
| 11022 | TDRKH | | |
| 100499489 | LOC100499489 | 4,371228857 | 6,3483E-05 |
| 969 | CD69 | 4,28849451 | 2,76355E-16 |
| 100506159 | LINC02470 | 4,226200057 | 4,52741E-05 |
| 100287559 | ADPGK-AS1 | 3,959016783 | 9,39096E-32 |
| 2865 | FFAR3 | 3,953616005 | 0,001879292 |
| 94234 | FOXQ1 | 3,827724334 | 0,002254717 |
| 91775 | NXPE3 | 3,895866798 | 4,2571E-27 |
| 399 | RHOH | 3,781312736 | 4,81064E-06 |
| 7079 | TIMP4 | 3,797426088 | 0,039300206 |
| 10538 | BATF | 3,799832446 | 1,87798E-16 |
| 3142 | HLX | 3,679761352 | 4,4754E-12 |
| 101927243 | LOC101927243 | 3,586415794 | 7,41159E-05 |
| 5621 | PRNP | 3,52824126 | 1,78921E-20 |
| 101927730 | LOC101927730 | | |
| 7052 | TGM2 | 3,338358446 | 7,96545E-06 |
| 127943 | FCRLB | | |
| 105373280 | NA | 3,84222075 | 1,09001E-11 |
| 6854 | SYN2 | 3,360968605 | 3,09182E-45 |
| 51339 | DACT1 | 3,149404521 | 3,70527E-05 |
| 406991 | MIR21 | 3,186606612 | 3,27801E-22 |
| 101928433 | ATXN1-AS1 | 2,96382782 | 1,6817E-42 |
| 80010 | RMI1 | 3,019166384 | 9,01519E-17 |
| 102724020 | LOC102724020 | 2,798583505 | 0,012773773 |
| 301 | ANXA1 | 2,918461352 | 3,06916E-12 |
| 50856 | CLEC4A | 2,849183076 | 4,08478E-22 |

| | | | |
|---|---|---|---|
| 27233 | SULT1C4 | 2,752499222 | 4,06591E-12 |
| 1906 | EDN1 | 2,780135194 | 1,8557E-08 |
| 8767 | RIPK2 | 2,792927394 | 1,27219E-18 |
| 114789 | SLC25A25 | 2,812497467 | 1,946E-07 |
| 105373989 | LOC105373989 | | |
| 89858 | SIGLEC12 | 2,641310069 | 0,018094321 |
| 89890 | KBTBD6 | 2,719449237 | 0,007860557 |
| 57559 | STAMBPL1 | 2,57898925 | 0,000224085 |
| 100505573 | INAFM2 | 2,527242136 | 1,27076E-24 |
| 152195 | NUDT16L2P | 2,619208605 | 2,22656E-31 |
| 23101 | MCF2L2 | 2,608685126 | 0,016907477 |
| 55862 | ECHDC1 | 2,551336921 | 1,33548E-10 |
| 79598 | CEP97 | 2,489272152 | 1,09883E-13 |
| 283587 | LINC01146 | 2,731730388 | 0,010890566 |
| 642559 | POU5F1P3 | 2,750022456 | 0,000986405 |
| 401409 | RAB19 | 2,536078769 | 0,000687576 |
| 2152 | F3 | 2,553901206 | 0,000162333 |
| 165140 | OXER1 | 2,611254404 | 9,64577E-19 |
| 56670 | SUCNR1 | 2,535620966 | 9,76001E-08 |
| 7678 | ZNF124 | 2,668799153 | 4,93496E-15 |
| 101929709 | RIPK2-DT | 2,477439908 | 1,83619E-08 |
| 100874309 | EPN2-IT1 | 2,24743365 | 0,007667941 |
| 5806 | PTX3 | 2,460648817 | 8,53549E-14 |
| 727993 | PDYN-AS1 | 2,217960148 | 0,00129099 |
| 100129405 | MSTO2P | 2,447778886 | 9,19318E-05 |
| 101927157 | LOC101927157 | 2,345665138 | 0,048827626 |
| 407006 | MIR221 | 2,450457104 | 0,003080725 |
| 101929623 | LINC01215 | 2,312361038 | 1,0335E-10 |
| 120939 | TMEM52B | 2,321515342 | 4,09836E-13 |
| 105371730 | LOC105371730 | 2,420413365 | 0,000560233 |
| 5608 | MAP2K6 | 2,375392537 | 4,66019E-09 |
| 440077 | ZNF705A | 2,853664379 | 0,001294485 |
| 83877 | TM2D2 | 2,330986916 | 3,00262E-57 |
| 339874 | NUDT16-DT | 2,225673026 | 0,00144278 |
| 123720 | WHAMM | 2,267665843 | 8,31459E-19 |
| 101927323 | PPP4R1-AS1 | 2,013735378 | 3,41513E-06 |
| 90459 | ERI1 | 2,196711065 | 0,001474577 |
| 440078 | FAM66C | 2,244254896 | 1,80461E-05 |
| 160364 | CLEC12A | 2,392324518 | 3,14305E-12 |
| 8843 | HCAR3 | 2,222161548 | 5,4443E-05 |
| 693120 | MIR33B | | |
| 407007 | MIR222 | 2,087288469 | 0,045141469 |
| 2992 | GYG1 | 2,285639786 | 7,98306E-07 |
| 93587 | TRMT10A | 2,213291266 | 6,04507E-06 |
| 10531 | PITRM1 | 2,129883056 | 3,47712E-06 |
| 9134 | CCNE2 | 2,076990839 | 0,005614548 |
| 9447 | AIM2 | 2,100890154 | 0,000507027 |
| 101927931 | LINC01480 | 2,065524014 | 0,010046873 |
| 123879 | DCUN1D3 | 1,939602045 | 0,001153304 |
| 6509 | SLC1A4 | 1,8180418 | 0,003857815 |
| 4973 | OLR1 | 1,975240515 | 5,65012E-14 |
| 388115 | CCDC9B | 2,041785485 | 3,07469E-13 |

| | | | |
|---|---|---|---|
| 51633 | OTUD6B | 1,866982538 | 1,16473E-07 |
| 29126 | CD274 | 2,043792174 | 0,011878321 |
| 10184 | LHFPL2 | 1,996155106 | 0,009342199 |
| 7846 | TUBA1A | 1,953452183 | 2,79594E-11 |
| 2210 | FCGR1B | 1,882159644 | 0,001346302 |
| 57116 | ZNF695 | 1,556550809 | 0,000570197 |
| 8195 | MKKS | 1,934524506 | 0,006921953 |
| 333932 | H3C15 | | |
| 23657 | SLC7A11 | 1,756798128 | 0,012997129 |
| 3625 | INHBB | 1,818119628 | 0,0003202 |
| 1027 | CDKN1B | 1,900739152 | 0,000134876 |
| 1435 | CSF1 | 1,805926837 | 0,009411106 |
| 51655 | RASD1 | 2,073303014 | 0,009411106 |
| 91754 | NEK9 | 1,825112429 | 1,11512E-12 |
| 100132417 | FCGR1CP | 1,728282456 | 0,003263904 |
| 10370 | CITED2 | 1,805982848 | 0,00026677 |
| 3552 | IL1A | 1,804470649 | 0,000932398 |
| 10376 | TUBA1B | 1,759130947 | 2,76355E-16 |
| 200058 | NA | 1,83646503 | 7,09454E-12 |
| 2209 | FCGR1A | 1,830487746 | 0,005370453 |
| 200261 | LINC00656 | 1,820175553 | 0,000114925 |
| 607 | BCL9 | 1,82831514 | 1,98647E-09 |
| 338442 | HCAR2 | 1,768485204 | 0,002184081 |
| 339929 | LPP-AS2 | 1,450412083 | 0,001047623 |
| 100170841 | EPOP | 1,421176388 | 0,002025855 |
| 5142 | PDE4B | 1,75714596 | 5,40875E-07 |
| 65010 | SLC26A6 | 1,699669903 | 2,62117E-07 |
| 79912 | PYROXD1 | 1,930596085 | 0,000371773 |
| 10435 | CDC42EP2 | 1,672802664 | 4,00319E-06 |
| 79746 | ECHDC3 | 1,702967058 | 6,59211E-05 |
| 57561 | ARRDC3 | 1,735673036 | 0,000610726 |
| 55728 | N4BP2 | 1,472958669 | 0,024776272 |
| 23401 | FRAT2 | 1,691931403 | 1,23122E-05 |
| 8477 | GPR65 | 1,633308736 | 1,08215E-05 |
| 90362 | FAM110B | 1,793348794 | 0,012680431 |
| 84790 | TUBA1C | 1,631205701 | 4,23516E-11 |
| 79660 | PPP1R3B | 1,637211335 | 2,02694E-06 |
| 7273 | TTN | 2,010771614 | 0,000142445 |
| 1389 | CREBL2 | 1,692476901 | 1,17493E-11 |
| 11103 | KRR1 | 1,648528238 | 4,27544E-05 |
| 337875 | H2BP1 | | |
| 337873 | H2BC20P | 1,716188653 | 2,63631E-09 |
| 113277 | TMEM106A | 1,498867894 | 0,0001734 |
| 340120 | ANKRD34B | 1,710016378 | 0,011257967 |
| 93474 | ZNF670 | 1,550077595 | 0,005370453 |
| 353189 | SLCO4C1 | 1,567390319 | 0,000333618 |
| 96626 | LIMS3 | 1,308000834 | 0,019330761 |
| 55972 | SLC25A40 | 1,352601892 | 0,003972251 |
| 84524 | ZC3H8 | 1,480834856 | 0,00838674 |
| 58500 | ZNF250 | 1,520377861 | 7,69713E-05 |
| 100303491 | ZEB2-AS1 | | |
| 65057 | ACD | 1,456081564 | 5,50598E-10 |

| | | | |
|---|---|---|---|
| 51651 | PTRH2 | 1,386975037 | 0,022671589 |
| 165186 | TOGARAM2 | 1,547354432 | 0,001492122 |
| 9560 | CCL4L2 | 1,381140378 | 0,001075983 |
| 2921 | CXCL3 | 1,49155488 | 0,009704073 |
| 5328 | PLAU | 1,373670677 | 0,045125836 |
| 10806 | SDCCAG8 | 1,403217542 | 0,001978546 |
| 2919 | CXCL1 | 1,416073715 | 0,013246068 |
| 57570 | TRMT5 | | |
| 10653 | SPINT2 | 1,334728502 | 0,042724976 |
| 100130476 | WAKMAR2 | 1,414103957 | 0,000224085 |
| 23528 | ZNF281 | 1,395328367 | 8,56909E-06 |
| 8797 | TNFRSF10A | 1,31198405 | 0,003685413 |
| 100507144 | LOC100507144 | | |
| 1477 | CSTF1 | 1,302822345 | 1,01538E-06 |
| 29886 | SNX8 | 1,390148648 | 0,002025094 |
| 7494 | XBP1 | 1,252785914 | 1,03197E-05 |
| 293 | SLC25A6 | 1,302281931 | 0,00032794 |
| 105371405 | NA | 1,304239212 | 0,000265269 |
| 54893 | MTMR10 | 1,309046594 | 7,17268E-09 |
| 6790 | AURKA | 1,323876698 | 4,00319E-06 |
| 100134229 | KDM7A-DT | 1,164081824 | 0,012997129 |
| 5768 | QSOX1 | 1,285827941 | 0,001043913 |
| 100506365 | OTUD6B-AS1 | 1,578227196 | 0,001978546 |
| 8337 | H2AC18 | | |
| 80231 | TASL | 1,264376072 | 0,002784065 |
| 8645 | KCNK5 | 1,216809676 | 2,77586E-05 |
| 169200 | TMEM64 | 1,155202184 | 0,002611723 |
| 8370 | H4C14 | 1,125109102 | 0,007272923 |
| 1647 | GADD45A | 1,147988559 | 0,004042941 |
| 2355 | FOSL2 | 1,108174423 | 0,000986405 |
| 9424 | KCNK6 | 1,032125323 | 0,019170611 |
| 57091 | CASS4 | 1,153634852 | 2,89859E-05 |
| 90355 | MACIR | | |
| 84777 | DLGAP1-AS2 | 1,082754392 | 0,017820135 |
| 414236 | C10orf55 | | |
| 112597 | CYTOR | 1,071676625 | 0,046988831 |
| 5734 | PTGER4 | 1,066071626 | 9,15918E-05 |
| 6458 | SH3GL1P1 | 1,235901592 | 0,001397854 |
| 83861 | RSPH3 | 1,235018313 | 3,41513E-06 |
| 79666 | PLEKHF2 | 1,003701462 | 1,24742E-09 |
| 91782 | CHMP7 | 0,960277484 | 0,048643996 |
| 28991 | COMMD5 | 1,030640425 | 0,0125525 |
| 1497 | CTNS | 1,136835751 | 0,001810922 |
| 127544 | RNF19B | 0,973147483 | 0,010231411 |
| 10289 | EIF1B | 1,013096617 | 1,38313E-07 |
| 23150 | FRMD4B | 1,005567945 | 0,0003202 |
| 6526 | SLC5A3 | | |
| 2280 | FKBP1A | 1,002947878 | 9,0262E-06 |
| 51768 | TM7SF3 | 0,962120611 | 0,009339173 |
| 204 | AK2 | 1,000049403 | 0,000735991 |
| 902 | CCNH | 0,905336523 | 0,009755877 |
| 79641 | ROGDI | 1,087563732 | 0,00014371 |

| | | | |
|---|---|---|---|
| 843 | CASP10 | 1,056570567 | 0,001474577 |
| 604 | BCL6 | 0,921935687 | 0,007579993 |
| 10018 | BCL2L11 | 1,012062986 | 0,008808883 |
| 124923 | RSKR | 1,072957711 | 4,02901E-05 |
| 290 | ANPEP | 0,934313378 | 0,00025678 |
| 115110 | TNFRSF14-AS1 | 0,963374013 | 0,019330761 |
| 23270 | TSPYL4 | 0,978032974 | 0,000731429 |
| 79145 | CHCHD7 | 0,85931008 | 8,25162E-08 |
| 2282 | FKBP1AP1 | | |
| 6310 | ATXN1 | 0,847453677 | 1,33032E-05 |
| 100507321 | ERVK13-1 | 1,00144376 | 0,006284853 |
| 734 | OSGIN2 | 0,904692976 | 3,51554E-10 |
| 23139 | MAST2 | | |
| 4783 | NFIL3 | 0,854035309 | 0,000181844 |
| 79865 | TREML2 | 0,821527078 | 0,001492122 |
| 64092 | SAMSN1 | 0,787072713 | 0,000195717 |
| 64783 | RBM15 | 0,708094449 | 0,000638672 |
| 353345 | GPR141 | 0,734319843 | 0,019961256 |
| 84984 | CEP19 | 0,670804009 | 0,000188327 |
| 9367 | RAB9A | 0,81777879 | 0,001667649 |
| 3587 | IL10RA | 0,778993148 | 0,003857815 |
| 6993 | DYNLT1 | 0,687665442 | 0,041586883 |
| 79042 | TSEN34 | 0,704059067 | 0,040653095 |
| 65264 | UBE2Z | | |
| 5868 | RAB5A | 0,675985566 | 6,7707E-05 |
| 83440 | ADPGK | 0,739630696 | 0,000618053 |
| 170688 | NUDT4P2 | 0,715513065 | 0,009342199 |
| 51406 | NOL7 | 0,765204308 | 0,000351331 |
| 440672 | NUDT4B | 0,653522991 | 0,002204634 |
| 7384 | UQCRC1 | 0,632006817 | 0,000568905 |
| 200298 | LINC00528 | 0,650543173 | 0,039300206 |
| 476 | ATP1A1 | | |
| 203245 | NAIF1 | 0,666204753 | 0,000932398 |
| 51765 | STK26 | 0,58812647 | 0,022783162 |
| 4245 | MGAT1 | 0,585812145 | 0,004216695 |
| 4648 | MYO7B | | |
| 51122 | COMMD2 | 0,507472187 | 0,015239111 |
| 9595 | CYTIP | 0,59069438 | 0,02199904 |
| 3066 | HDAC2 | 0,465783021 | 0,009796502 |
| 23710 | GABARAPL1 | 0,54135816 | 0,0201391 |
| 84124 | ZNF394 | 0,606946195 | 0,004315764 |
| 2850 | GPR27 | | |
| 7259 | TSPYL1 | 0,558892761 | 0,001244623 |
| 11163 | NUDT4 | | |
| 9839 | ZEB2 | 0,487444284 | 0,011875514 |
| 3554 | IL1R1 | 0,437802292 | 0,038049084 |
| 137835 | TMEM71 | 0,432310585 | 0,007667941 |
| 55350 | VNN3 | 0,484939918 | 0,005676511 |
| 51465 | UBE2J1 | 0,445847367 | 0,009704073 |
| 55716 | LMBR1L | | |
| 57414 | RHBDD2 | 0,446944747 | 0,000178275 |
| 29116 | MYLIP | 0,359874153 | 0,005422274 |

| | | | |
|---|---|---|---|
| 79571 | GCC1 | | |
| 4050 | LTB | -0,302184052 | 0,033977259 |
| 54386 | TERF2IP | | |
| 5606 | MAP2K3 | -0,396838257 | 0,006284853 |
| 2117 | ETV3 | -0,44058956 | 0,002891442 |
| 6195 | RPS6KA1 | -0,42147783 | 6,04507E-06 |
| 754 | PTTG1IP | -0,390490953 | 0,039788853 |
| 7559 | ZNF12 | -0,508238263 | 0,001047623 |
| 51043 | ZBTB7B | -0,502592557 | 0,001796127 |
| 10970 | CKAP4 | -0,529396239 | 0,00370073 |
| 1627 | DBN1 | -0,545815665 | 0,010293926 |
| 262 | AMD1 | -0,497752188 | 4,65136E-05 |
| 64114 | TMBIM1 | -0,565788866 | 0,000845236 |
| 90007 | MIDN | -0,643476078 | 0,000224085 |
| 84967 | LSM10 | -0,618091858 | 0,005752783 |
| 9181 | ARHGEF2 | -0,648597711 | 0,0125525 |
| 4615 | MYD88 | -0,644666181 | 0,004640757 |
| 84225 | ZMYND15 | -0,606082804 | 0,028650756 |
| 93129 | ORAI3 | -0,779467128 | 0,009411106 |
| 1241 | LTB4R | | |
| 100505783 | OSER1-DT | | |
| 79768 | KATNBL1 | -0,811585511 | 0,011160378 |
| 284702 | NA | -0,798998378 | 0,014050836 |
| 7532 | YWHAG | -0,813285743 | 0,014189195 |
| 220359 | TIGD3 | | |
| 100505869 | CFAP58-DT | -0,816595561 | 0,015246045 |
| 11344 | TWF2 | | |
| 29946 | SERTAD3 | -0,811732364 | 0,030333955 |
| 56905 | C15orf39 | -0,879338436 | 0,004444802 |
| 27113 | BBC3 | -1,05021222 | 3,1355E-05 |
| 8013 | NR4A3 | -1,0506789 | 0,00370073 |
| 126364 | LRRC25 | -0,908632712 | 0,000312809 |
| 27173 | SLC39A1 | -0,902610577 | 2,60168E-06 |
| 91869 | RFT1 | -0,991393188 | 0,014189195 |
| 144811 | LACC1 | -0,822887967 | 0,038457807 |
| 3638 | INSIG1 | -1,077625473 | 0,00025678 |
| 29948 | OSGIN1 | -1,032217786 | 0,01556384 |
| 10294 | DNAJA2 | -1,04602584 | 0,027500444 |
| 23601 | CLEC5A | -1,328703743 | 0,002255414 |
| 85450 | ITPRIP | -1,076535287 | 0,005291587 |
| 2876 | GPX1 | -1,151039052 | 0,019330761 |
| 284029 | LINC00324 | -1,118732608 | 0,013341397 |
| 65108 | MARCKSL1 | -1,276127912 | 0,00029427 |
| 113540 | CMTM1 | -1,447181782 | 9,97407E-05 |
| 103752588 | PACERR | -1,231666453 | 0,016062849 |
| 148304 | C1orf74 | -1,420696341 | 3,64568E-05 |
| 133 | ADM | -1,325995704 | 0,042724976 |
| 54101 | RIPK4 | -1,46309465 | 0,021264606 |
| 8740 | TNFSF14 | -1,468376958 | 0,009530609 |
| 7071 | KLF10 | -1,445637104 | 0,034190713 |
| 643155 | SMIM15 | -1,380589612 | 0,018560201 |
| 101927472 | ITPRIP-AS1 | -1,479424509 | 0,00025678 |

| | | | |
|---|---|---|---|
| 102465451 | MIR6753 | | |
| 375307 | CATIP | -1,543081417 | 0,018534372 |
| 9935 | MAFB | -1,995324536 | 0,006147841 |
| 388 | RHOB | -1,999574862 | 0,003368022 |
| 1306 | COL15A1 | | |
| 3593 | IL12B | -1,753874084 | 0,00099839 |
| 54541 | DDIT4 | -2,632375171 | 2,97972E-11 |
| 7124 | TNF | -2,85332432 | 1,83879E-07 |
| 3976 | LIF | -3,471109855 | 6,63039E-09 |
| 6615 | SNAI1 | -4,208273691 | 1,52632E-05 |
| 3627 | CXCL10 | | |
| 116071 | BATF2 | | |
| 92421 | CHMP4C | | |
| 730249 | ACOD1 | | |
| 6355 | CCL8 | | |
| 83480 | PUS3 | | |
| 105371932 | LOC105371932 | | |
| 22928 | SEPHS2 | | |
| 100287902 | LGALS8-AS1 | | |
| 115004 | CGAS | | |
| 1073 | CFL2 | | |
| 389073 | C2orf80 | | |
| 64375 | IKZF4 | | |
| 3872 | KRT17 | | |
| 5414 | SEPTIN4 | | |
| 26784 | SNORA64 | | |
| 56833 | SLAMF8 | | |
| 389289 | ANXA2R | | |
| 92797 | HELB | | |
| 677844 | SNORA78 | | |
| 57102 | C12orf4 | | |
| 92610 | TIFA | | |
| 80832 | APOL4 | | |
| 574042 | SNORA10 | | |
| 56169 | GSDMC | | |
| 3433 | IFIT2 | | |
| 3659 | IRF1 | | |
| 7993 | UBXN8 | | |
| 9021 | SOCS3 | | |
| 400759 | GBP1P1 | | |
| 731424 | MIR3945HG | | |
| 55853 | IDI2-AS1 | | |
| 2622 | GAS8 | | |
| 56829 | ZC3HAV1 | | |
| 5464 | PPA1 | | |
| 83636 | C19orf12 | | |
| 8542 | APOL1 | | |
| 55863 | TMEM126B | | |
| 2633 | GBP1 | | |
| 27198 | HCAR1 | | |
| 735301 | SNHG9 | | |
| 26262 | TSPAN17 | | |

| | | | |
|---|---|---|---|
| 10161 | LPAR6 | | |
| 3399 | ID3 | | |
| 79898 | ZNF613 | | |
| 114294 | LACTB | | |
| 2730 | GCLM | | |
| 26270 | FBXO6 | | |
| 102465879 | MIR8085 | | |
| 51513 | ETV7 | | |
| 55748 | CNDP2 | | |
| 10613 | ERLIN1 | | |
| 100873924 | HLX-AS1 | | |
| 2635 | GBP3 | | |
| 80830 | APOL6 | | |
| 1847 | DUSP5 | | |
| 55435 | AP1AR | | |
| 23283 | CSTF2T | | |
| 101928674 | SOCS3-DT | | |
| 100101490 | MCTS2P | | |
| 84290 | CAPNS2 | | |
| 84549 | MAK16 | | |
| 8482 | SEMA7A | | |
| 2531 | KDSR | | |
| 6890 | TAP1 | | |
| 285966 | TCAF2 | | |
| 27074 | LAMP3 | | |
| 6737 | TRIM21 | | |
| 100500818 | MIR3945 | | |
| 3665 | IRF7 | | |
| 154761 | LOC154761 | | |
| 124460 | SNX20 | | |
| 129531 | MITD1 | | |
| 6741 | SSB | | |
| 56061 | UBFD1 | | |
| 100131998 | RRN3P3 | | |
| 57147 | SCYL3 | | |
| 399972 | GSEC | | |
| 101927780 | LINC01303 | | |
| 28512 | NKIRAS1 | 1,178193746 | 0,035473323 |
| 100129354 | NRADDP | | |
| 151636 | DTX3L | | |
| 105221694 | BISPR | | |
| 79689 | STEAP4 | | |
| 339541 | ARMH1 | | |
| 246754 | MTVR2 | | |
| 6897 | TARS1 | | |
| 7752 | ZNF200 | | |
| 51118 | UTP11 | | |
| 27309 | ZNF330 | | |
| 27071 | DAPP1 | | |
| 100506229 | LINC01093 | | |
| 54968 | TMEM70 | | |
| 54957 | TXNL4B | | |

| | |
|---|---|
| 115362 | GBP5 |
| 100507331 | ZSWIM8-AS1 |
| 51734 | MSRB1 |
| 10906 | TRAFD1 |
| 7855 | FZD5 |
| 83452 | RAB33B |
| 122769 | LRR1 |
| 25939 | SAMHD1 |
| 51311 | TLR8 |
| 23172 | ABRAXAS2 |
| 639 | PRDM1 |
| 9603 | NFE2L3 |
| 10623 | POLR3C |
| 10749 | KIF1C |
| 4055 | LTBR |
| 3964 | LGALS8 |
| 9674 | KIAA0040 |
| 79134 | TMEM185B |
| 6773 | STAT2 |
| 102724552 | RERE-AS1 |
| 548645 | DNAJC25 |
| 3383 | ICAM1 |
| 211 | ALAS1 |
| 3759 | KCNJ2 |
| 80124 | VCPIP1 |
| 26118 | WSB1 |
| 23557 | SNAPIN |
| 29978 | UBQLN2 |
| 688 | KLF5 |
| 7706 | TRIM25 |
| 3428 | IFI16 |
| 51263 | MRPL30 |
| 5709 | PSMD3 |
| 6187 | RPS2 |
| 57172 | CAMK1G |
| 8804 | CREG1 |
| 7132 | TNFRSF1A |
| 100419583 | LOC100419583 |
| 9416 | DDX23 |
| 29097 | CNIH4 |
| 54149 | C21orf91 |
| 2956 | MSH6 |
| 9489 | PGS1 |
| 85363 | TRIM5 |
| 9754 | STARD8 |
| 2146 | EZH2 |
| 23780 | APOL2 |
| 117289 | TAGAP |
| 1390 | CREM |
| 113419 | TEX261 |
| 6398 | SECTM1 |
| 1439 | CSF2RB |

| | | | |
|---|---|---|---|
| 84674 | CARD6 | | |
| 57018 | CCNL1 | | |
| 5696 | PSMB8 | | |
| 6609 | SMPD1 | | |
| 51303 | FKBP11 | | |
| 54623 | PAF1 | | |
| 11277 | TREX1 | | |
| 5936 | RBM4 | | |
| 132720 | FAM241A | | |
| 4678 | NASP | | |
| 64422 | ATG3 | | |
| 400617 | KCNJ2-AS1 | | |
| 11040 | PIM2 | | |
| 10261 | IGSF6 | | |
| 90410 | IFT20 | | |
| 22904 | SBNO2 | | |
| 11017 | SNRNP27 | | |
| 2867 | FFAR2 | | |
| 3422 | IDI1 | | |
| 8509 | NDST2 | | |
| 197259 | MLKL | | |
| 1992 | SERPINB1 | | |
| 54665 | RSBN1 | | |
| 10423 | CDIPT | | |
| 55741 | EDEM2 | | |
| 7126 | TNFAIP1 | | |
| 56929 | FEM1C | | |
| 55969 | RAB5IF | | |
| 10865 | ARID5A | | |
| 105372417 | NA | | |
| 79713 | IGFLR1 | | |
| 23433 | RHOQ | | |
| 6203 | RPS9 | | |
| 91748 | MIDEAS | | |
| 602 | BCL3 | | |
| 9344 | TAOK2 | | |
| 26229 | B3GAT3 | | |
| 2634 | GBP2 | | |
| 81602 | CDADC1 | | |
| 57862 | ZNF410 | | |
| 84248 | FYTTD1 | | |
| 64127 | NOD2 | | |
| 29107 | NXT1 | | |
| 3320 | HSP90AA1 | | |
| 9221 | NOLC1 | | |
| 11025 | LILRB3 | | |
| 8737 | RIPK1 | | |
| 2242 | FES | | |
| 79155 | TNIP2 | | |
| 6921 | ELOC | | |
| 353274 | ZNF445 | | |
| 100507463 | PSMB8-AS1 | 0,617447281 | 0,04365932 |

| | | | |
|---|---|---|---|
| 7572 | ZNF24 | | |
| 55096 | EBLN2 | | |
| 1856 | DVL2 | | |
| 7913 | DEK | | |
| 57532 | NUFIP2 | | |
| 3708 | ITPR1 | | |
| 5777 | PTPN6 | | |
| 5970 | RELA | | |
| 55588 | MED29 | | |
| 54556 | ING3 | | |
| 55048 | VPS37C | | |
| 55911 | APOBR | | |
| 152302 | CIDECP1 | | |
| 80213 | TM2D3 | -0,47990058 | 0,041405398 |
| 11212 | PLPBP | | |
| 51177 | PLEKHO1 | | |
| 9659 | PDE4DIP | | |
| 124599 | CD300LB | | |
| 3312 | HSPA8 | | |
| 58160 | NFE4 | | |
| 6446 | SGK1 | | |
| 7852 | CXCR4 | | |
| 79697 | RIOX1 | | |
| 171177 | RHOV | | |
| 200942 | KLHDC8B | | |
| 7621 | ZNF70 | | |
| 135228 | CD109 | 4,781884237 | 0,046843159 |
| 284018 | C17orf58 | 2,182596878 | 0,037958462 |
| 347902 | AMIGO2 | 2,154329082 | 0,016430309 |
| 10224 | ZNF443 | 2,102745982 | 0,013885749 |
| 91523 | PCED1B | 2,07733858 | 0,035473323 |
| 5347 | PLK1 | 1,776604723 | 0,017612097 |
| 10924 | SMPDL3A | 1,767162094 | 0,0125525 |
| 8704 | B4GALT2 | 1,736494228 | 0,020026593 |
| 10276 | NET1 | 1,612166361 | 0,015857551 |
| 284323 | ZNF780A | 1,277755007 | 0,01656656 |
| 3624 | INHBA | 1,215989609 | 0,019961256 |
| 65986 | ZBTB10 | 1,185302297 | 0,045141469 |
| 10943 | MSL3 | 0,903425801 | 0,033516533 |
| 285237 | C3orf38 | 0,899746181 | 0,006471517 |
| 57149 | LYRM1 | 0,800200331 | 0,045100081 |
| 5226 | PGD | 0,776929852 | 0,019961256 |
| 649446 | DLGAP1-AS1 | 0,751555004 | 0,014531358 |
| 169693 | TMEM252 | 0,73893776 | 0,027500444 |
| 92667 | MGME1 | 0,534218411 | 0,026548172 |
| 139716 | GAB3 | 0,531174012 | 0,005422274 |
| 4778 | NFE2 | 0,471392732 | 0,045437294 |
| 1982 | EIF4G2 | 0,323742184 | 0,046843159 |
| 4296 | MAP3K11 | -0,354636512 | 0,013533054 |
| 382 | ARF6 | -0,377710747 | 0,003445433 |
| 1968 | EIF2S3 | -0,422804739 | 0,031649099 |
| 8778 | SIGLEC5 | -0,544246703 | 0,045141469 |

| | | | |
|---|---|---|---|
| 10127 | ZNF263 | -0,593113469 | 0,004443957 |
| 30834 | POLR1H | -0,689377072 | 0,003624784 |
| 4795 | NFKBIL1 | -0,753141099 | 0,005669527 |
| 6804 | STX1A | -0,761826236 | 0,04792402 |
| 1774 | DNASE1L1 | -0,790193446 | 0,045125836 |
| 729176 | KATNBL1P6 | -0,796513234 | 0,045100081 |
| 221710 | SMIM13 | -0,798518289 | 0,005185139 |
| 55194 | EVA1B | -0,818854647 | 0,003751598 |
| 197320 | ZNF778 | -0,986153435 | 0,030122874 |
| 285193 | DUSP28 | -1,312317813 | 0,000786893 |
| 2828 | GPR4 | -1,74376632 | 0,02199904 |
| 100616284 | MIR4489 | -1,81684863 | 0,016946213 |
| 100996539 | ITPR1-DT | -1,910331752 | 0,017612097 |
| 388610 | TRNP1 | -4,67575431 | 0,024706066 |
| | | | |

| IL-13 stimulation | | IFNg stimulation | |
|---|---|---|---|
| **log2foldchange2** | **padj2** | **log2FoldChange3** | **padj3** |
| 8,346007089 | 1,05306E-10 | | |
| 7,560420486 | 1,00444E-56 | 6,445739237 | 1,48865E-40 |
| 7,277892822 | 1,40151E-08 | 4,81184702 | 0,002559793 |
| 7,124240749 | 1,28389E-17 | | |
| 6,455274091 | 1,34925E-49 | 3,538023148 | 9,97175E-14 |
| 6,22304545 | 1,6666E-12 | | |
| 6,118700195 | 0,00013706 | 4,391003902 | 0,034134543 |
| 5,761411123 | 0,03891878 | | |
| 5,397435754 | 7,22263E-17 | 2,477577204 | 0,003612705 |
| 5,313866621 | 0,000188001 | | |
| 5,261402968 | 0,001156684 | | |
| 5,167540262 | 1,54491E-52 | | |
| 5,166403585 | 0,000333725 | | |
| 5,1282076 | 0,00165049 | | |
| 4,981681015 | 1,02848E-06 | | |
| 4,932436935 | 0,00232357 | | |
| 4,915418632 | 1,28389E-17 | | |
| 4,829527352 | 0,007697048 | | |
| 4,78781146 | 0,00978087 | 4,269975501 | 0,046119496 |
| 4,626596962 | 8,70052E-33 | 2,734643385 | 1,23709E-10 |
| 4,51812263 | 0,026252937 | | |
| 4,501713885 | 1,36726E-15 | | |
| 4,453651681 | 0,040494585 | | |
| 4,285629144 | 0,00010625 | | |
| 4,174890401 | 2,10163E-15 | | |
| 4,127248774 | 8,15993E-05 | | |
| 3,925308653 | 3,10758E-31 | | |
| 3,919217228 | 0,002291863 | 3,223347757 | 0,035144384 |
| 3,915531061 | 0,00167841 | | |
| 3,848688175 | 1,89314E-26 | | |
| 3,763590537 | 5,53601E-06 | 3,100136006 | 0,000781864 |
| 3,729679371 | 0,048245054 | | |
| 3,700342671 | 1,42299E-15 | 3,727115403 | 4,12979E-15 |
| 3,603526697 | 1,59177E-11 | 3,294840645 | 3,30636E-09 |
| 3,597595712 | 7,22252E-05 | | |
| 3,555285362 | 8,25168E-21 | | |
| 3,555122709 | 0,04783422 | | |
| 3,513733083 | 1,68547E-06 | | |
| 3,413284012 | 0,03597737 | | |
| 3,408373713 | 5,10411E-09 | 2,393079682 | 0,000469304 |
| 3,375267237 | 1,29777E-45 | | |
| 3,139913676 | 3,98661E-05 | | |
| 3,115359675 | 3,55316E-21 | 1,52588259 | 0,000166044 |
| 3,02849643 | 1,90162E-44 | | |
| 2,987798251 | 2,05736E-16 | | |
| 2,958034818 | 0,00670762 | | |
| 2,898271784 | 4,42215E-12 | | |
| 2,859043804 | 3,04201E-22 | | |

| | | | |
|---|---|---|---|
| 2,824292699 | 8,94309E-13 | | |
| 2,821615993 | 1,10384E-08 | | |
| 2,789917057 | 1,49341E-18 | | |
| 2,776843519 | 3,11498E-07 | | |
| 2,763259371 | 0,021312359 | | |
| 2,76288296 | 0,011274354 | 4,60251162 | 1,66407E-07 |
| 2,746946681 | 0,007343484 | | |
| 2,708592655 | 8,01907E-05 | | |
| 2,630157362 | 9,53489E-27 | | |
| 2,619080735 | 2,69786E-31 | | |
| 2,614077468 | 0,018162924 | | |
| 2,603611282 | 4,98281E-11 | | |
| 2,597001825 | 5,88486E-15 | | |
| 2,567852099 | 0,024737521 | | |
| 2,563750324 | 0,003491691 | | |
| 2,545169574 | 0,000685693 | | |
| 2,528003742 | 0,00020596 | | |
| 2,49026058 | 5,45578E-17 | | |
| 2,485142709 | 2,1234E-07 | | |
| 2,484623836 | 6,73317E-13 | | |
| 2,47880028 | 1,77312E-08 | | |
| 2,461759903 | 0,002018904 | | |
| 2,443444909 | 1,33578E-13 | | |
| 2,413035442 | 0,000284133 | | |
| 2,39215628 | 0,000161744 | | |
| 2,391736722 | 0,042346966 | | |
| 2,389108905 | 0,004765241 | | |
| 2,371528397 | 3,02847E-11 | | |
| 2,36562119 | 1,28864E-13 | | |
| 2,344921778 | 0,001053481 | | |
| 2,318860169 | 1,39148E-08 | | |
| 2,307560499 | 0,02735818 | | |
| 2,30429673 | 8,64371E-56 | | |
| 2,287355657 | 0,000959357 | | |
| 2,281118608 | 4,82601E-19 | | |
| 2,272486054 | 4,37494E-08 | | |
| 2,25957378 | 0,000975513 | | |
| 2,251647135 | 1,70869E-05 | | |
| 2,230328186 | 1,47286E-10 | | |
| 2,184789838 | 8,32803E-05 | 1,997455306 | 0,000781864 |
| 2,184605918 | 0,040318566 | | |
| 2,132623983 | 0,03891878 | | |
| 2,131163836 | 6,8954E-06 | | |
| 2,101745273 | 2,53183E-05 | | |
| 2,093373772 | 5,68617E-06 | 1,43501069 | 0,012166392 |
| 2,066175116 | 0,006601811 | | |
| 2,036568136 | 0,000944837 | | |
| 2,027680141 | 0,013301827 | | |
| 2,006979295 | 0,000679078 | 1,76384549 | 0,00687373 |
| 2,005973168 | 0,000836424 | | |
| 2,002811779 | 2,12418E-14 | | |
| 1,996000897 | 1,30648E-12 | | |

| | | | |
|---|---|---|---|
| 1,962167923 | 1,52858E-08 | | |
| 1,932969771 | 0,024387225 | 3,393502007 | 3,0391E-07 |
| 1,925434842 | 0,015162835 | | |
| 1,890362639 | 1,58016E-10 | | |
| 1,885467626 | 0,001377648 | 3,100050908 | 9,00806E-10 |
| 1,884998519 | 4,99509E-06 | | |
| 1,882051561 | 0,010266009 | | |
| 1,881752837 | 0,004530074 | | |
| 1,840228586 | 0,007703514 | | |
| 1,838717859 | 0,000273989 | | |
| 1,816593959 | 0,000349852 | 3,943758285 | 3,85828E-13 |
| 1,804211256 | 0,00978087 | | |
| 1,804064034 | 0,046098978 | | |
| 1,800777314 | 2,44868E-12 | | |
| 1,799775953 | 0,001785362 | 3,338368496 | 4,04688E-12 |
| 1,784994263 | 0,000350579 | | |
| 1,784578309 | 0,001174104 | | |
| 1,777478775 | 1,36067E-16 | 0,770861151 | 0,008132556 |
| 1,777311202 | 4,58545E-11 | | |
| 1,771776553 | 0,008579921 | 3,324769498 | 6,44324E-10 |
| 1,766238126 | 0,000228726 | | |
| 1,759087391 | 1,16645E-08 | | |
| 1,753527812 | 0,002672146 | 1,912450548 | 0,0008707 |
| 1,734572989 | 2,41806E-05 | | |
| 1,72547808 | 4,32972E-05 | | |
| 1,718524421 | 1,14237E-06 | 1,306615309 | 0,001293831 |
| 1,710334043 | 2,19031E-07 | | |
| 1,693540946 | 0,003888749 | | |
| 1,685722409 | 3,24672E-06 | | |
| 1,683555918 | 8,86782E-05 | | |
| 1,682927413 | 0,00111009 | | |
| 1,679312083 | 0,005415168 | | |
| 1,65653528 | 2,20719E-05 | | |
| 1,651395824 | 8,02788E-06 | | |
| 1,650898575 | 0,034699678 | | |
| 1,627941605 | 4,98281E-11 | | |
| 1,623580926 | 2,6067E-06 | 1,599260014 | 7,11178E-06 |
| 1,607202166 | 0,007626657 | | |
| 1,586736782 | 3,81421E-10 | | |
| 1,586297002 | 0,000110638 | | |
| 1,557384976 | 0,011815298 | | |
| 1,535118458 | 2,6179E-07 | | |
| 1,514792038 | 0,000138666 | | |
| 1,50708904 | 0,04638028 | | |
| 1,506068158 | 0,008396219 | | |
| 1,503846395 | 0,000798906 | | |
| 1,503445428 | 0,003491691 | | |
| 1,49643627 | 0,000836424 | | |
| 1,494521188 | 0,007703514 | | |
| 1,494407381 | 0,000118531 | | |
| 1,458543906 | 0,03891878 | | |
| 1,458326099 | 5,31393E-10 | | |

| | | | |
|---|---|---|---|
| 1,434781466 | 0,017161718 | | |
| 1,430921132 | 0,005579647 | | |
| 1,430878189 | 0,000614709 | | |
| 1,413651952 | 0,020024004 | | |
| 1,402018788 | 0,03891878 | | |
| 1,39239398 | 0,002373344 | | |
| 1,38202184 | 0,019248793 | | |
| 1,363921018 | 0,018035043 | | |
| 1,358486435 | 0,038536854 | | |
| 1,354333165 | 0,000559543 | | |
| 1,349171406 | 2,16604E-05 | | |
| 1,343409936 | 0,002710839 | | |
| 1,326492886 | 0,013186598 | | |
| 1,321836897 | 6,17109E-07 | | |
| 1,317930059 | 0,004919445 | | |
| 1,29720484 | 3,84272E-06 | | |
| 1,293411802 | 0,000395301 | | |
| 1,293032705 | 0,000333374 | | |
| 1,284301495 | 1,64629E-08 | | |
| 1,275707599 | 1,23644E-05 | | |
| 1,274320798 | 0,004312337 | | |
| 1,273005455 | 0,001300283 | | |
| 1,23738677 | 0,043360543 | | |
| 1,220721246 | 0,03891878 | | |
| 1,191006591 | 0,00702077 | 1,407220004 | 0,000696115 |
| 1,178246997 | 6,27428E-05 | | |
| 1,170743068 | 0,002254854 | | |
| 1,163785691 | 0,004918696 | | |
| 1,162509715 | 0,003629699 | | |
| 1,151611616 | 0,000513808 | | |
| 1,148164777 | 0,005556092 | | |
| 1,141489964 | 3,72359E-05 | | |
| 1,1413688 | 0,048314049 | | |
| 1,13135926 | 0,011274354 | | |
| 1,115451846 | 0,025361419 | | |
| 1,112897282 | 0,035401769 | | |
| 1,107859022 | 3,72359E-05 | | |
| 1,100178015 | 0,008633603 | | |
| 1,07680722 | 0,000125159 | | |
| 1,074061528 | 4,58545E-11 | | |
| 1,068617422 | 0,017170303 | | |
| 1,036194084 | 0,012278003 | | |
| 1,025296791 | 0,008579921 | | |
| 1,020687255 | 0,005954797 | | |
| 1,007723876 | 1,77872E-07 | | |
| 1,006900614 | 0,000333374 | 0,913361595 | 0,002561357 |
| 1,002187733 | 0,009813908 | | |
| 0,987019896 | 1,42722E-05 | | |
| 0,985952843 | 0,007160819 | | |
| 0,97752323 | 0,001156684 | | |
| 0,961466875 | 0,004756803 | | |
| 0,957850944 | 0,001719234 | | |

| | | | |
|---|---|---|---|
| 0,956996245 | 0,007038246 | | |
| 0,933825998 | 0,006841577 | | |
| 0,930641874 | 0,024737521 | | |
| 0,925489248 | 0,000968987 | | |
| 0,920690389 | 0,000350579 | | |
| 0,907702167 | 0,039406632 | | |
| 0,899117212 | 0,003168703 | | |
| 0,897486762 | 1,40523E-08 | | |
| 0,878618056 | 0,022025846 | | |
| 0,874916717 | 5,53601E-06 | | |
| 0,86585711 | 0,038054454 | | |
| 0,865220909 | 3,13268E-09 | | |
| 0,825634809 | 0,001505774 | 0,663243288 | 0,032977728 |
| 0,816915525 | 0,000455043 | | |
| 0,809112024 | 0,002030395 | | |
| 0,774137332 | 0,000284133 | | |
| 0,768412533 | 0,000128709 | | |
| 0,740265273 | 0,020342615 | | |
| 0,732980421 | 2,41806E-05 | -0,665048718 | 0,000446939 |
| 0,731458817 | 0,008984706 | -0,706322144 | 0,024670966 |
| 0,729946225 | 0,009741923 | | |
| 0,70755153 | 0,033621805 | | |
| 0,704679065 | 0,041466206 | 1,147528987 | 1,69673E-05 |
| 0,699526567 | 0,024737521 | | |
| 0,69930168 | 3,00131E-05 | | |
| 0,697517279 | 0,001777459 | | |
| 0,696724755 | 0,013427151 | | |
| 0,672847221 | 0,003593422 | | |
| 0,665791335 | 0,001754178 | | |
| 0,657934512 | 0,000273989 | | |
| 0,650706812 | 0,040535536 | | |
| 0,623961021 | 0,033415632 | | |
| 0,617186111 | 0,003491691 | | |
| 0,592627692 | 0,023396545 | | |
| 0,585563635 | 0,00462613 | | |
| 0,58038702 | 0,024737521 | | |
| 0,574951262 | 0,003141228 | | |
| 0,565648599 | 0,038515526 | | |
| 0,548681869 | 0,000898459 | | |
| 0,528321686 | 0,02923254 | | |
| 0,519713354 | 0,031145779 | | |
| 0,509695139 | 0,001622182 | | |
| 0,506353668 | 0,006060485 | | |
| 0,500200494 | 0,035724203 | | |
| 0,46640633 | 0,021312359 | | |
| 0,465970143 | 0,021062907 | | |
| 0,431599427 | 0,008236019 | | |
| 0,420538077 | 0,033621805 | | |
| 0,413631254 | 0,024737521 | | |
| 0,395816163 | 0,043221206 | | |
| 0,369770521 | 0,005093748 | | |
| 0,30228979 | 0,042406663 | | |

| | | | |
|---|---|---|---|
| -0,284254484 | 0,042408306 | -0,336883883 | 0,007539284 |
| -0,309498183 | 0,028413326 | | |
| -0,315791365 | 0,040704768 | | |
| -0,378465196 | 0,012124027 | | |
| -0,392408657 | 0,014189192 | | |
| -0,403660551 | 2,02433E-05 | | |
| -0,436174979 | 0,012278003 | | |
| -0,444245869 | 0,008256423 | -0,402910681 | 0,03068751 |
| -0,475208455 | 0,004606872 | | |
| -0,519076624 | 0,005252755 | | |
| -0,563091402 | 0,007472334 | | |
| -0,567136787 | 1,2791E-06 | | |
| -0,58862866 | 0,00042042 | | |
| -0,602482482 | 0,000836424 | | |
| -0,609587209 | 0,007472334 | | |
| -0,622720455 | 0,021529714 | -0,632919011 | 0,022099323 |
| -0,627173082 | 0,007274976 | | |
| -0,676078499 | 0,008579921 | | |
| -0,687795282 | 0,039560776 | | |
| -0,690124898 | 0,049256055 | | |
| -0,697321943 | 0,045174331 | | |
| -0,713153176 | 0,04656584 | | |
| -0,73237359 | 0,03891878 | | |
| -0,73878879 | 0,042099444 | | |
| -0,782638311 | 0,03463665 | | |
| -0,79855667 | 0,021505311 | | |
| -0,800729439 | 0,049984527 | | |
| -0,810484645 | 0,033621805 | | |
| -0,839402522 | 0,008633603 | | |
| -0,852961603 | 0,002005468 | | |
| -0,864519742 | 0,040312075 | | |
| -0,89061393 | 0,000482392 | | |
| -0,891555863 | 3,79124E-06 | | |
| -0,92708675 | 0,033022774 | | |
| -0,956076086 | 0,007703514 | | |
| -0,994433545 | 0,001182196 | | |
| -1,003498782 | 0,023413297 | | |
| -1,023323208 | 0,037894036 | | |
| -1,043703745 | 0,044389722 | | |
| -1,065546721 | 0,006546193 | | |
| -1,085836956 | 0,03891878 | | |
| -1,141096549 | 0,011274354 | | |
| -1,195452064 | 0,001052419 | | |
| -1,264151071 | 0,001326827 | | |
| -1,283912789 | 0,010430239 | | |
| -1,339371411 | 0,000135728 | -0,976573199 | 0,020192559 |
| -1,346025626 | 0,03891878 | | |
| -1,386100608 | 0,040312075 | | |
| -1,387465217 | 0,020109881 | | |
| -1,404545969 | 0,046504857 | | |
| -1,438436298 | 0,012357704 | | |
| -1,49399375 | 0,000228525 | | |

| | | | |
|---|---|---|---|
| -1,558933864 | 0,019369459 | | |
| -1,734331635 | 0,004756803 | | |
| -1,787099058 | 0,024737521 | | |
| -1,879801279 | 0,008238422 | | |
| -1,947738905 | 0,040704768 | | |
| -1,990160319 | 0,000128709 | -1,504149158 | 0,008025443 |
| -2,543026752 | 1,82511E-10 | | |
| -2,816521593 | 2,97002E-07 | | |
| -3,1211125 | 4,27079E-07 | | |
| -4,279447786 | 1,00476E-05 | | |
| | | 7,318494891 | 1,34523E-06 |
| | | 5,863834042 | 9,97175E-14 |
| | | 5,767923674 | 0,002833484 |
| | | 5,304932855 | 0,034605503 |
| | | 5,295111586 | 0,034134543 |
| | | 4,928525151 | 0,001784094 |
| | | 4,795846811 | 0,00848188 |
| | | 4,788676762 | 4,77722E-23 |
| | | 4,725358931 | 0,001533623 |
| | | 4,540955142 | 2,84166E-19 |
| | | 4,4647567 | 1,75879E-10 |
| | | 4,246151164 | 0,006290785 |
| | | 4,246145134 | 4,30365E-21 |
| | | 4,172159225 | 1,20215E-10 |
| | | 4,125922406 | 0,021734504 |
| | | 4,050053151 | 1,29592E-07 |
| | | 3,75424803 | 0,034423916 |
| | | 3,686517511 | 3,28334E-09 |
| | | 3,542533422 | 1,56018E-07 |
| | | 3,539304555 | 4,29967E-05 |
| | | 3,503922134 | 7,59717E-12 |
| | | 3,445893279 | 9,27239E-12 |
| | | 3,210031702 | 0,002316688 |
| | | 3,160492416 | 0,002787755 |
| | | 3,127726832 | 0,007591275 |
| | | 3,118196457 | 0,002088513 |
| | | 3,068338409 | 3,85828E-13 |
| | | 3,065367336 | 0,00512186 |
| | | 2,895213757 | 1,06564E-05 |
| | | 2,883288091 | 0,003238512 |
| | | 2,844012796 | 5,25227E-09 |
| | | 2,621462174 | 0,035380792 |
| | | 2,581345414 | 6,96219E-07 |
| | | 2,542170672 | 1,66607E-05 |
| | | 2,536850265 | 2,96012E-05 |
| | | 2,506596188 | 1,90838E-05 |
| | | 2,492723868 | 1,06564E-05 |
| | | 2,476278522 | 6,88925E-11 |
| | | 2,47359307 | 0,000173158 |
| | | 2,473145612 | 0,000505833 |
| | | 2,443396923 | 4,92674E-09 |
| | | 2,431379864 | 2,72113E-05 |

| | |
|---|---|
| 2,377960838 | 0,006323533 |
| 2,365331833 | 0,012886253 |
| 2,333805549 | 0,046119496 |
| 2,306212944 | 2,57027E-11 |
| 2,267146827 | 6,06158E-06 |
| 2,262311872 | 1,47164E-05 |
| 2,257562152 | 0,014992987 |
| 2,251466936 | 0,000462214 |
| 2,237137389 | 0,003612705 |
| 2,223368597 | 3,79543E-06 |
| 2,191469576 | 0,04956243 |
| 2,188975966 | 0,002276958 |
| 2,173613279 | 0,001575857 |
| 2,16353593 | 1,22474E-05 |
| 2,160660906 | 6,67309E-08 |
| 2,156760181 | 6,74052E-08 |
| 2,15645846 | 1,47164E-05 |
| 2,147873078 | 0,003168523 |
| 2,145688805 | 0,005502173 |
| 2,14118315 | 0,014701781 |
| 2,102695788 | 0,028626695 |
| 2,098972761 | 6,46401E-07 |
| 2,091350704 | 1,21216E-07 |
| 2,080284318 | 0,000545833 |
| 2,078766477 | 0,008479139 |
| 2,074906296 | 8,29782E-06 |
| 2,040879478 | 0,000110523 |
| 2,02952442 | 7,20273E-08 |
| 2,005933143 | 0,000397864 |
| 2,001344221 | 1,59843E-08 |
| 1,992942777 | 6,06158E-06 |
| 1,927754855 | 5,78016E-05 |
| 1,90300412 | 0,028057366 |
| 1,901188323 | 0,000143632 |
| 1,895425248 | 3,79466E-05 |
| 1,868483036 | 0,000529006 |
| 1,847721827 | 8,012E-05 |
| 1,846193695 | 2,68116E-05 |
| 1,840984597 | 7,20273E-08 |
| 1,833834871 | 0,000934949 |
| 1,832183087 | 0,002734893 |
| 1,832070201 | 0,002303422 |
| 1,799908126 | 0,02398321 |
| 1,778394429 | 0,007539284 |
| 1,732848705 | 5,82657E-06 |
| 1,732217521 | 0,000725033 |
| 1,71783902 | 0,003064024 |
| 1,694692751 | 1,23249E-05 |
| 1,667367916 | 0,001395147 |
| 1,651159703 | 0,039905073 |
| 1,640175884 | 4,75856E-05 |
| 1,627457855 | 1,46862E-06 |

| | |
|---|---|
| 1,619477726 | 0,024350118 |
| 1,61468234 | 0,000128291 |
| 1,61396796 | 8,95512E-07 |
| 1,611613291 | 0,006214051 |
| 1,608121346 | 0,041674491 |
| 1,607897891 | 2,8556E-09 |
| 1,602145607 | 0,004486297 |
| 1,586277954 | 0,01524591 |
| 1,577087015 | 2,22851E-05 |
| 1,574609213 | 0,000507861 |
| 1,569338055 | 0,024350118 |
| 1,561333798 | 0,02133345 |
| 1,560441183 | 0,006058235 |
| 1,559399062 | 8,64462E-05 |
| 1,548490948 | 0,001239965 |
| 1,54156281 | 0,005466723 |
| 1,538193941 | 9,58946E-05 |
| 1,531696797 | 0,007152716 |
| 1,52901044 | 0,001395147 |
| 1,523595198 | 0,005659609 |
| 1,52319008 | 3,32829E-05 |
| 1,521411054 | 0,032949081 |
| 1,479644721 | 1,17427E-05 |
| 1,471636729 | 0,000797204 |
| 1,469182774 | 0,000725033 |
| 1,450287939 | 0,002834829 |
| 1,433904426 | 0,000555065 |
| 1,422114965 | 1,12807E-08 |
| 1,40986952 | 0,000225355 |
| 1,402817156 | 0,000295046 |
| 1,39863408 | 0,003182887 |
| 1,397540056 | 0,007295335 |
| 1,386902876 | 0,000414412 |
| 1,386356637 | 1,48895E-07 |
| 1,378255187 | 0,005788108 |
| 1,358183632 | 0,002013774 |
| 1,329750051 | 0,007591275 |
| 1,315036577 | 0,000497885 |
| 1,312373663 | 0,016182113 |
| 1,305178973 | 0,01392789 |
| 1,305174225 | 0,010497871 |
| 1,287510401 | 0,016159204 |
| 1,286231739 | 0,013995724 |
| 1,278480561 | 0,00311037 |
| 1,266144937 | 0,001296371 |
| 1,264796499 | 0,023207194 |
| 1,25604546 | 0,003412332 |
| 1,250473449 | 0,0019339 |
| 1,242309911 | 0,003305048 |
| 1,241252809 | 0,010828042 |
| 1,222125246 | 0,012166392 |
| 1,221051428 | 0,003360936 |

| | |
|---|---|
| 1,178004859 | 0,011750078 |
| 1,175540164 | 0,014873138 |
| 1,174745769 | 0,003238512 |
| 1,173887473 | 0,000464828 |
| 1,166474076 | 0,001724459 |
| 1,162970366 | 0,001109724 |
| 1,156742563 | 0,046525802 |
| 1,137218507 | 6,05925E-05 |
| 1,098855412 | 0,004240959 |
| 1,097312782 | 0,000785997 |
| 1,076983184 | 0,018097942 |
| 1,070053981 | 0,029578124 |
| 1,057766881 | 0,003344841 |
| 1,037473243 | 0,043199198 |
| 1,02191161 | 0,049381254 |
| 1,021786848 | 0,001123547 |
| 1,019609455 | 0,014199816 |
| 1,019247259 | 0,0056254 |
| 1,01743172 | 2,72113E-05 |
| 1,013712217 | 0,033046302 |
| 1,008051057 | 0,003324455 |
| 1,000617516 | 0,012260165 |
| 0,994757436 | 0,000128291 |
| 0,978304218 | 0,001328607 |
| 0,97702381 | 0,008481299 |
| 0,968724967 | 0,035510173 |
| 0,962515133 | 0,006287397 |
| 0,941654075 | 0,02082045 |
| 0,935174123 | 0,004486297 |
| 0,934797648 | 0,025006978 |
| 0,933398641 | 0,034134543 |
| 0,925351051 | 0,007295335 |
| 0,919498469 | 0,000667499 |
| 0,918276384 | 0,007792059 |
| 0,91776356 | 0,008256377 |
| 0,909722843 | 0,001774551 |
| 0,888218731 | 0,01005934 |
| 0,882490705 | 0,048447907 |
| 0,866927332 | 0,024670966 |
| 0,839563024 | 0,014873138 |
| 0,832100474 | 0,012753192 |
| 0,82943115 | 0,049381254 |
| 0,82001128 | 0,011427329 |
| 0,804612661 | 0,034326781 |
| 0,798164889 | 0,017500006 |
| 0,769887494 | 0,01005934 |
| 0,71230164 | 0,026523936 |
| 0,702782155 | 0,011896782 |
| 0,700094051 | 0,000797204 |
| 0,697485981 | 0,000976453 |
| 0,675411828 | 0,015522422 |
| 0,624570845 | 0,04956243 |

| | | |
|---|---|---|
| | 0,579322949 | 0,027607283 |
| | 0,577558247 | 0,022099323 |
| | 0,563761162 | 0,0296956 |
| | 0,549837418 | 0,002013774 |
| | 0,538313367 | 0,024350118 |
| | 0,535859367 | 0,036716796 |
| | 0,497582329 | 0,046619791 |
| | 0,482424711 | 0,032139114 |
| | 0,467634188 | 0,042818401 |
| | 0,333829798 | 0,047599986 |
| | -0,353502628 | 0,005585562 |
| | -0,368825707 | 0,024160205 |
| | -0,430716686 | 0,012995068 |
| | -0,491812091 | 0,041442649 |
| | -0,536071487 | 0,001533623 |
| | -0,555674754 | 0,013949312 |
| | -0,733637817 | 0,048556645 |
| | -0,750592947 | 0,036716796 |
| | -0,777717166 | 0,007949533 |
| | -0,781258968 | 0,012166392 |
| | -0,809157669 | 0,019025371 |
| | -1,107640699 | 0,001025074 |
| | -1,309258651 | 0,049381254 |
| | -1,446193301 | 0,019165817 |
| | -1,46375083 | 0,002280977 |
| | -1,661152059 | 0,049462895 |

| **Comparison** | **Adjusted p-value** |
|---|---|
| Unstim vs. IL-4 0.01 ng/ml | 0.0073 |
| Unstim vs. il-4 0.1 ng/ml | 0.001 |
| Unstim vs. IL-4 1 ng/ml | 0.001 |
| Unstim vs. IL-4 10 ng/ml | 0.0044 |
| Unstim vs. IL-4 100 ng/ml | 0.0004 |
| Unstim vs. IL-13 0.01 ng/ml | 0.9998 |
| Unstim vs. IL-13 0.1 ng/ml | 0.0545 |
| Unstim vs. IL-13 1 ng/ml | 0.0015 |
| Unstim vs. IL-13 10 ng/ml | 0.0008 |
| Unstim vs. IL-13 100 ng/ml | 0.0003 |

Supplementary Table 3. Adjusted p-values of indicated IL-4 and IL-13 stimulations in human neutrophils, calculated by ANOVA with Dunnett’s multiple comparison test.
